# TaME-seq: An efficient sequencing approach for characterisation of HPV genomic variability and chromosomal integration

**DOI:** 10.1038/s41598-018-36669-6

**Published:** 2019-01-24

**Authors:** Sonja Lagström, Sinan Uğur Umu, Maija Lepistö, Pekka Ellonen, Roger Meisal, Irene Kraus Christiansen, Ole Herman Ambur, Trine B. Rounge

**Affiliations:** 1 Department of Microbiology and Infection Control, Akershus University Hospital, Lørenskog, Norway; 2 Department of Research, Cancer Registry of Norway, Oslo, Norway; 3 Institute for Molecular Medicine Finland, University of Helsinki, Helsinki, Finland; 4 Clinical Molecular Biology (EpiGen), Medical Division, Akershus University Hospital and Institute of Clinical Medicine, University of, Oslo, Norway; 5 Faculty of Health Sciences, OsloMet - Oslo Metropolitan University, Oslo, Norway

## Abstract

HPV genomic variability and chromosomal integration are important in the HPV-induced carcinogenic process. To uncover these genomic events in an HPV infection, we have developed an innovative and cost-effective sequencing approach named TaME-seq (tagmentation-assisted multiplex PCR enrichment sequencing). TaME-seq combines tagmentation and multiplex PCR enrichment for simultaneous analysis of HPV variation and chromosomal integration, and it can also be adapted to other viruses. For method validation, cell lines (n = 4), plasmids (n = 3), and HPV16, 18, 31, 33 and 45 positive clinical samples (n = 21) were analysed. Our results showed deep HPV genome-wide sequencing coverage. Chromosomal integration breakpoints and large deletions were identified in HPV positive cell lines and in one clinical sample. HPV genomic variability was observed in all samples allowing identification of low frequency variants. In contrast to other approaches, TaME-seq proved to be highly efficient in HPV target enrichment, leading to reduced sequencing costs. Comprehensive studies on HPV intra-host variability generated during a persistent infection will improve our understanding of viral carcinogenesis. Efficient identification of both HPV variability and integration sites will be important for the study of HPV evolution and adaptability and may be an important tool for use in cervical cancer diagnostics.

## Introduction

Human papillomavirus (HPV) is the main cause of cervical cancer^1^, one of the most common cancers in women worldwide, causing more than 200,000 deaths each year^2,3^. A persistent infection with HPV high-risk genotypes is recognised as a necessary cause of cancer development^4^. Of the 13 carcinogenic high-risk types, HPV16 and 18 are associated with about 70% of all cervical cancers^5,6^. HPV infection is also associated with cancer in penis, vulva, vagina, anus, and head and neck^7^. However, only a small fraction of HPV infections at any site will progress to cancer^8^. This indicates that in addition to HPV infection, additional factors such as HPV genomic variability and integration, could contribute to the HPV-induced carcinogenic process. An appropriate sequencing approach is needed to uncover these genomic events during a persistent HPV infection.

HPV contains an approximately 7.9 kb circular double-stranded DNA genome, consisting of early region (E1, E2, E4-7) genes, late region (L1, L2) genes and an upstream regulatory region (URR)^9^. To date, more than 200 HPV types have been identified^10^. Each individual HPV type shares at least 90% sequence identity in the conserved L1 open reading frame (ORF) nucleotide sequence. Isolates of the same HPV types that differ by 1–10% or 0.5–1% across the genome are referred to as variant lineages or sublineages, respectively^11,12^.

Despite phylogenetic relatedness, HPV variant lineages can differ in their carcinogenic potential^13–16^. Traditionally, studies have focused on cancer risk of main variants. However, recent studies have revealed variability below the level of variant lineages that may be evidence of intra-host viral evolution and adaptation^17–20^. In contrast to a limited number of studies on HPV variability, HPV integration into the host genome has been more widely studied and is regarded as a determining event in cervical carcinogenesis^21–23^. Upon integration, disruption or complete deletion of the E1 or E2 gene is often observed, resulting in constitutive expression of the E6 and E7 oncogenes^24–26^, inactivation of cell cycle checkpoints and genetic instability^23^. Viral integration may also lead to modified expression of cellular genes nearby, disruption of genes, as well as genomic amplifications that may promote oncogenesis^23,27^. The finding of certain chromosomal clusters of integration in precancerous lesions and cancers^28^ also suggests a selective advantage of specific HPV integrations. Still, several important questions remain for HPV integration and more comprehensive analyses of integration sites are needed in order to expand our understanding of HPV pathogenesis.

The development of next generation sequencing (NGS) technologies has provided new tools for viral genomic research. During the recent years, a few studies have described different NGS based approaches to study HPV variability and integration in the human genome. The most common approaches used in HPV genomic analyses are based on target enrichment using highly multiplexed degenerate primers^29^, enrichment by multiplex PCR using HPV16 forward primers^30^, bead-based target capture^31–33^, and rolling circle amplification^34^ followed by NGS. These methods are however designed to detect either HPV integration or HPV variability. In addition, target capture methods poorly enrich HPV and remain expensive due to high probe cost and off-target sequencing.

**Figure 1:**
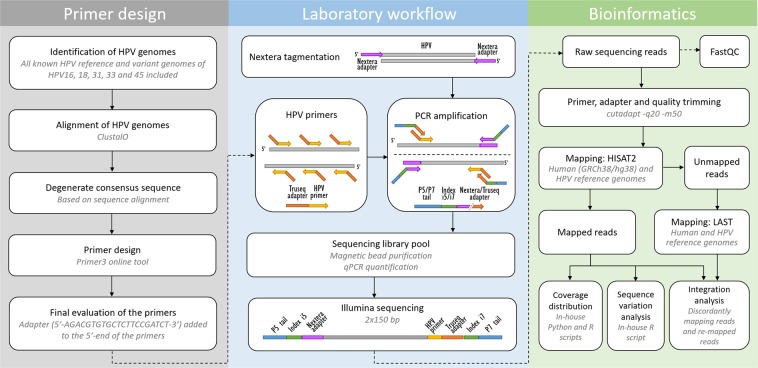
Primer design, laboratory and bioinformatics workflows of the TaME-seq method.

In order to contribute to the understanding of the role of intra-host HPV genomic variability and chromosomal integration in carcinogenesis, we have developed an innovative library preparation strategy followed by an in-house bioinformatics pipeline named TaME-seq (tagmentation-assisted multiplex PCR enrichment sequencing). TaME-seq combines tagmentation and multiplex PCR enrichment, allowing simultaneous HPV genomic variability and integration analysis (Fig. 1). TaME-seq, with highly efficient target enrichment and reduced sequencing cost, enables deep sequencing analysis in order to find low frequency variants and rare integration events. Here, we present the results of HPV integration and genomic variability analysis in HPV16, 18, 31, 33 and 45 positive clinical samples and cell lines. The method described here provides an important tool for comprehensive studies of HPV genomic variability and chromosomal integration, and it can also be adapted to studies on other viruses such as retroviruses, adeno-associated viruses and integrating human herpesviruses.

## Results

### Read mapping analysis and genome coverage

**Figure 2:**
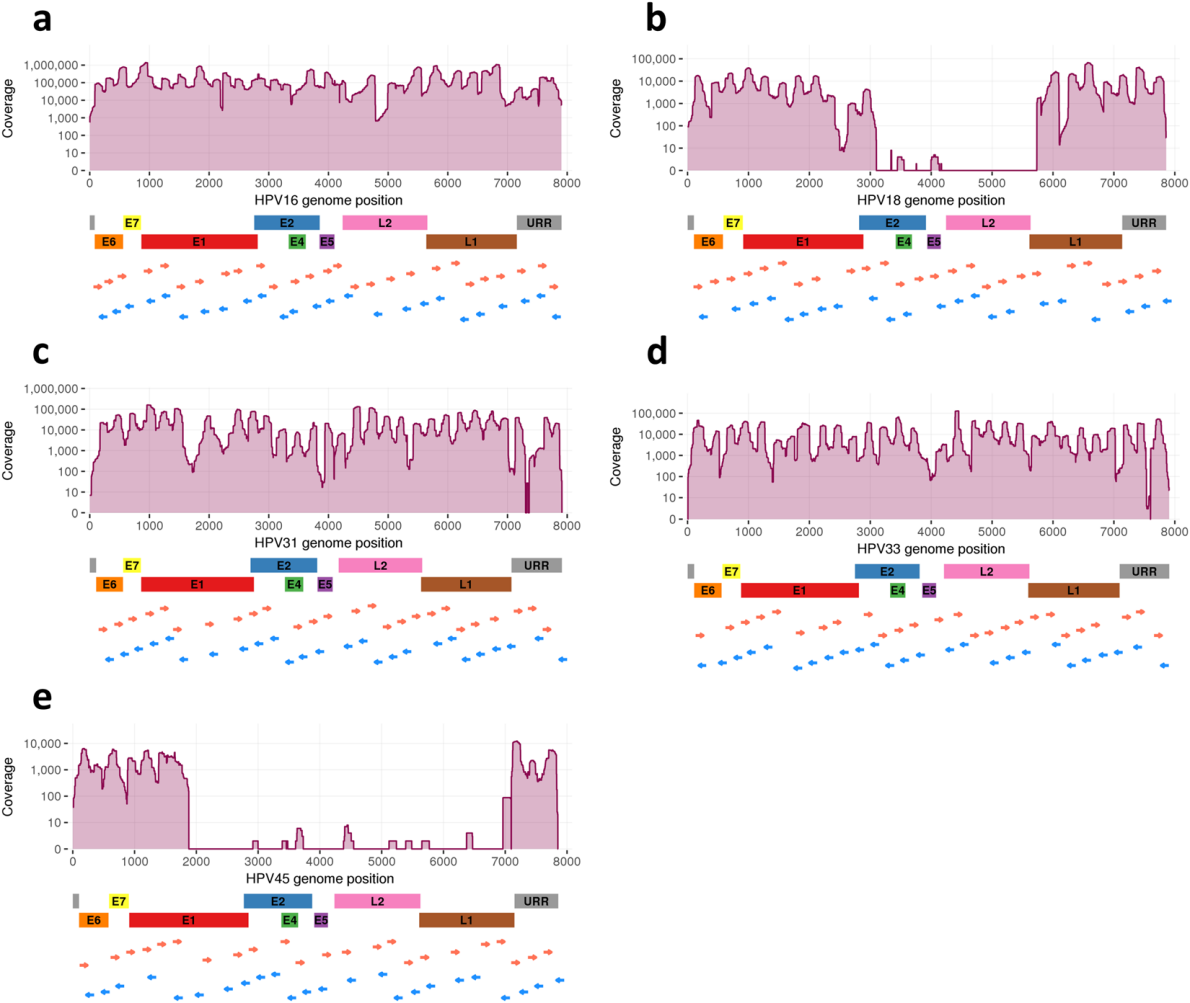
HPV genome sequencing coverage in HPV positive samples. The coverage plots of (**a**) CaSki, (**b**) HeLa, (**c**) LBC34, (**d**) LBC11, and (**e**) MS751 are aligned to the respective target HPV genomes. The location of early (E1, E2, E4-7), late (L1, L2) genes, URR, and forward (red arrows) and reverse (blue arrows) HPV primers is indicated below the genomic positions.

Table 1 summarises liquid-based cytology (LBC) samples (n = 21), cell lines (n = 4) and plasmid samples (n = 3) included in the analysis. The samples generated 154.8 million raw reads of which 72.5 million reads (47%) mapped to the target HPV reference genomes. Only a small fraction (0.08%) of the reads mapped to other HPV types than those reported positive by HPV genotyping. The mean coverage ranged from 303 to 273898, while the fraction of the genome covered by minimum 10 × ranged from 0.35 to 1, and the fraction of the genome covered by minimum 100 × ranged from 0.33 to 1 (Table 1). HPV genome sequencing coverage aligned to the target HPV genomes with the location of HPV genomic regions and primers is visualised for CaSki, HeLa, LBC34, LBC11 and MS751 (Fig. 2). Overall, the samples showed varying HPV genome coverage profiles (Supplementary Figs S1–S5). Totally, 10 HPV positive samples were excluded from further analysis due to poor sequencing coverage (Supplementary Table S1). Sequencing of the HPV negative control samples resulted in no or negligible amount (<500) of reads mapped to target HPV genomes (Supplementary Table S2). The MS751 cell line was confirmed not to contain HPV18 sequences (Supplementary Table S1)^35^.

**Table 1:** Read counts and sequencing coverage of HPV positive cell lines, plasmids and LBC samples.

Sample	Sample type	Raw reads	Trimmed reads	Reads mapped to target HPV	% Reads mapped to target HPV	Mean coverage	Fraction of genome covered by minimum	
							10×	100×
***HPV16***								
CaSki	Cell line	16138790^b^	12944262	12634651	78%	184716	1.00	1.00
SiHa	Cell line	151168^b^	133360	67496	45%	1018	0.96	0.83
SiHa-1	Cell line	5948008^c^	3735936	1249594	21%	17561	0.93	0.90
SiHa-1	Cell line	844178^b^	532874	181199	21%	2554	0.92	0.78
SiHa-2	Cell line	1405886^c^	789664	420774	30%	5609	0.91	0.85
SiHa-2	Cell line	158672^b^	90150	48412	31%	646	0.84	0.52
WHO std HPV16	Plasmid	359638^b^	304002	278987	78%	4104	0.99	0.96
LBC1^a^	LBC	128008^b^	108756	75323	59%	1124	0.96	0.88
LBC7^a^	LBC	62246^b^	51590	25567	41%	384	0.94	0.66
***HPV18***								
HeLa	Cell line	1433248^b^	1120824	394420	28%	5897	0.68	0.62
WHO std HPV18	Plasmid	2021206^b^	1358182	1098783	54%	15447	0.99	0.96
LBC103^a^	LBC	1477706^b^	1209564	74358	5%	1056	0.93	0.83
LBC105^a^	LBC	190664^b^	160450	32695	17%	484	0.51	0.34
LBC107	LBC	2180284^b^	1881868	978435	45%	14663	1.00	0.99
LBC108^a^	LBC	5407154^b^	3773986	3360463	62%	46691	1.00	0.98
LBC48^a^	LBC	641378^b^	433884	72589	11%	988	0.95	0.83
***HPV31***								
LBC16	LBC	276994^b^	191290	74465	27%	1065	0.94	0.80
LBC24^a^	LBC	471666^b^	348416	24197	5%	355	0.96	0.69
LBC32	LBC	2446832^b^	1523572	1319939	54%	18983	0.99	0.98
LBC34	LBC	3285680^b^	1841812	1723631	52%	23790	0.99	0.96
***HPV33***								
HPV33 plasmid	Plasmid	13824396^b^	5202718	5230090	38%	61527	1.00	1.00
LBC11	LBC	2852262^b^	1052512	986936	35%	12038	0.99	0.98
LBC30	LBC	77128^b^	51682	21431	28%	303	0.93	0.63
LBC31^a^	LBC	4276740^c^	2831408	44917	1.1%	544	0.76	0.60
LBC52	LBC	154936^b^	86990	34390	22%	439	0.95	0.62
LBC65^a^	LBC	368260^b^	248142	144022	39%	1993	1.00	0.91
***HPV45***								
MS751	Cell line	1221694^b^	1047286	56291	5%	845	0.35	0.33
LBC13^a^	LBC	496370^b^	389306	58293	12%	849	0.96	0.78
LBC29	LBC	211052^b^	122502	45925	22%	614	0.91	0.69
LBC36^a^	LBC	2412532^b^	1822912	1579570	65%	22093	1.00	0.97
LBC54	LBC	50169422^c^	26385910	20570184	41%	256857	1.00	1.00
LBC64^a^	LBC	5121416^c^	3040714	307476	6%	3943	0.95	0.88

^a^Sample has multiple HPV infections.

^b^Sequenced on MiSeq sequencing platform.

^c^Sequenced on HiSeq 2500 sequencing platform.

### Deletions in HPV genomes

The method enables identification of regions covered with very few or no sequencing reads, interpreted as large HPV genomic deletions. Cell lines HeLa and MS751 are known to contain partial HPV genomes due to deletions of 2.5 kb and 5 kb, respectively^35,36^, which was confirmed by our method (Fig. 2). A large deletion of 4.8 kb was revealed in the clinical sample LBC105, indicating partial or complete deletion of HPV18 genes E1, E2, E4, E5, L1 and L2 (Supplementary Fig. S2).

### HPV-human integration sites

**Figure 3:**
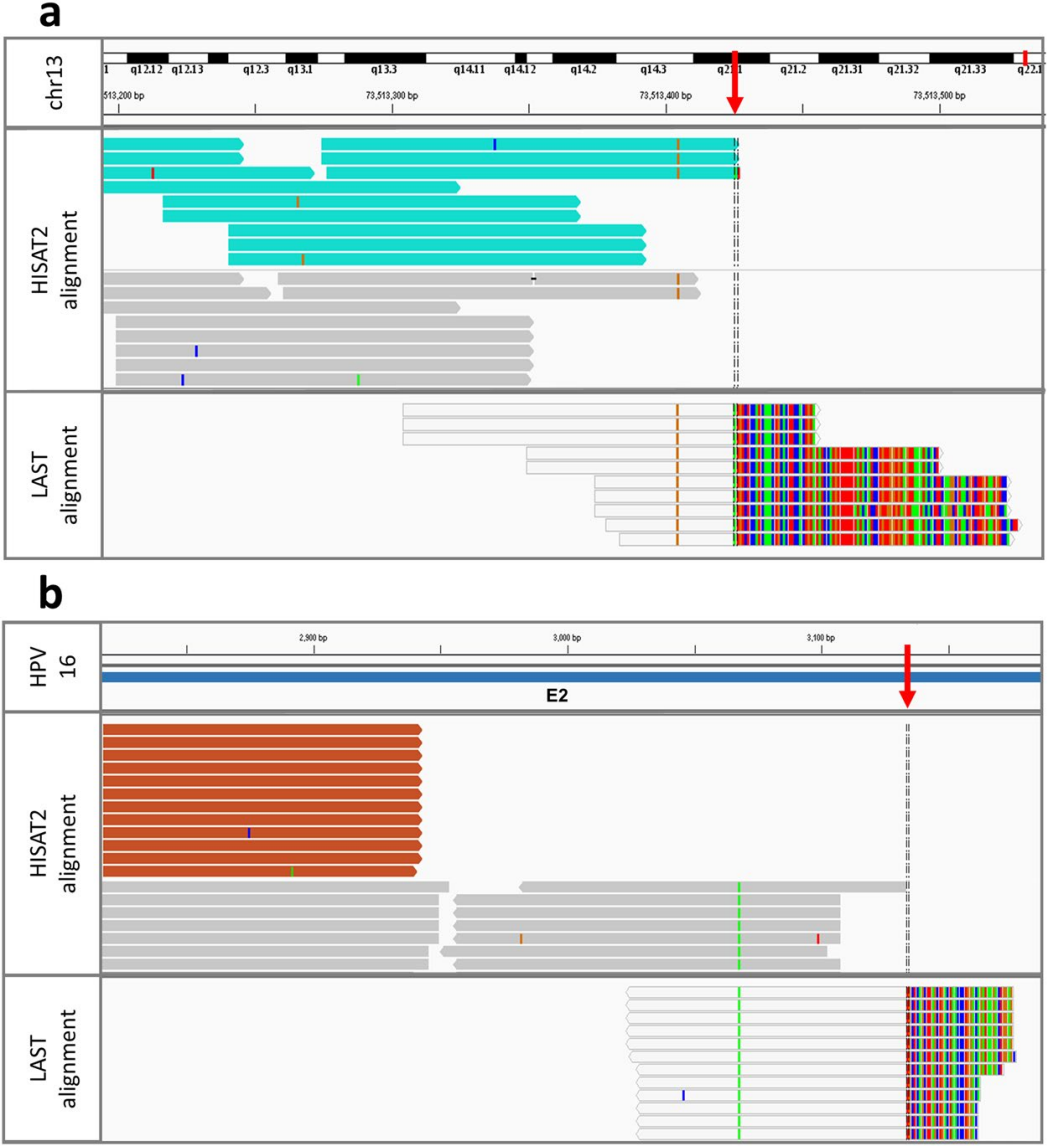
An IGV visualisation of HISAT2 and LAST alignments to find HPV-human integration breakpoints. All the reads were first mapped with HISAT2 and then the unmapped reads were remapped with LAST. (**a**) SiHa reads mapping to chromosome 13 (GRCh38/hg38). Light blue HISAT2 reads have pairs mapping to HPV16 reference genome. Multi-coloured parts of the LAST reads are mismatched bases that map to HPV16 (not visualised). (**b**) SiHa reads mapping to HPV16 reference genome. Orange HISAT2 reads have pairs mapping to chromosome 13 (GRCh38/hg38). Multi-coloured parts of the LAST reads are mismatched bases that map to chromosome 13 (not visualised). Red arrows point to the exact breakpoint positions.

A two-step strategy was applied to detect possible integration sites (Fig. 3). A total of 27 integration sites were detected in cell lines CaSki, SiHa, HeLa and MS571 (Table 2). For CaSki, 16 previously reported integration sites^30,32,37^ were confirmed. In addition, three novel sites were identified. These mapped to HPV16 E6, E2 and L1 genes. One was located in an intronic region of the gene *BRSK1*; two were located more than 50 kb from annotated genes (Table 2). Three sites, including one previously reported site as a control^30,37^, were subjected to Sanger sequencing to confirm the integration sites (Supplementary Table S3). Integration sites identified in SiHa, HeLa and MS751 were consistent with previous studies^31,35–39^ and were not subjected to validation by Sanger sequencing. Additionally, two integration sites were detected in the clinical sample LBC105 (Table 2). The integration breakpoints were mapped to the HPV E1 and L1 genes flanking the deleted region (Supplementary Fig. S2) and they were located in intronic regions of the gene *GTF2IRD1* (Table 2). Both integration sites were confirmed by Sanger sequencing (Supplementary Table S3).

**Table 2:** Chromosomal integration sites detected by TaME-seq.

Sample	HPV		Human (GRCh38/hg38)		# Unique discordant read pairs	# Unique junction reads
	Breakpoint	ORF	Chromosomal locus	Breakpoint		
***HPV16***						
CaSki	273	E6	20p11.1	chr20:26276796	19	0^e^
	494^a^	E6	20p11.1	chr20:26341342^b^	7	0^e^
	582	E7	19q13.42	chr19:55310208	0	15
	975	E1	Xq27.3	chrX:145696778	0	7
	1398	E1	2p23.3	chr2:27135968	6	0^e^
	1793	E1	10p14	chr10:11700197	4	0^e^
	2987	E2	Xq27.3	chrX:145708231	3	8
	3239	E2	7p22.1	chr7:6925283	5	0^e^
	3631^a^	E2	19q13.42	chr19:55310043^c^	3	0^e^
	3729	E2	6p21.1	chr6:45691388	0	11
	4654	L2	11p15.4	chr11:6741077	11	0^e^
	5432	L2	11q22.1	chr11:100766632	2	0^e^
	5698	L1	10p14	chr10:11700617	20	0^e^
	5698	L1	5p11	chr5:46292081	2	0^e^
	5762	L1	11q22.1	chr11:100771699	4	0^e^
	6572	L1	19q13.42	chr19:55307445	3	0^e^
	7123^a^	L1	20p11.1	chr20:26357640^b^	20	0^e^
	7733	URR	11p15.4	chr11:6740842	2	0^e^
	7733	URR	2p23.3	chr2:27137265	6	0^e^
SiHa	3133	E2	13q22.1	chr13:73513425	7	7
	3385	E2/E4	13q22.1	chr13:73214729	3	0^e^
***HPV18***						
HeLa	2066	E1	8q24.21	chr8:127229053	2	0^e^
	2887	E2	8q24.21	chr8:127221122	13	0^e^
	5730	L1	8q24.21	chr8:127218384	11	89
	7655	URR	8q24.21	chr8:127221804	3	0^e^
LBC105	1561	E1	7q11.23	chr7:74525628^d^	0	10
LBC105	6528	L1	7q11.23	chr7:74515883^d^	2	0^e^
***HPV45***						
MS751	1646	E1	18q11.2	chr18:23024744	10	0^e^
	7120	L1	18q11.2	chr18:23021388	15	0^e^

^a^Novel breakpoint in CaSki cell line.

^b^No annotated genes within 50 kb from the breakpoint.

^c^Intronic region in gene *BRSK1*.

^d^Intronic region in gene *GTF2IRD1*.

^e^When number of unique junction reads is 0, the breakpoint coordinates are not exact.

### Evaluation of variant calling using SiHa technical replicates

**Figure 4:**
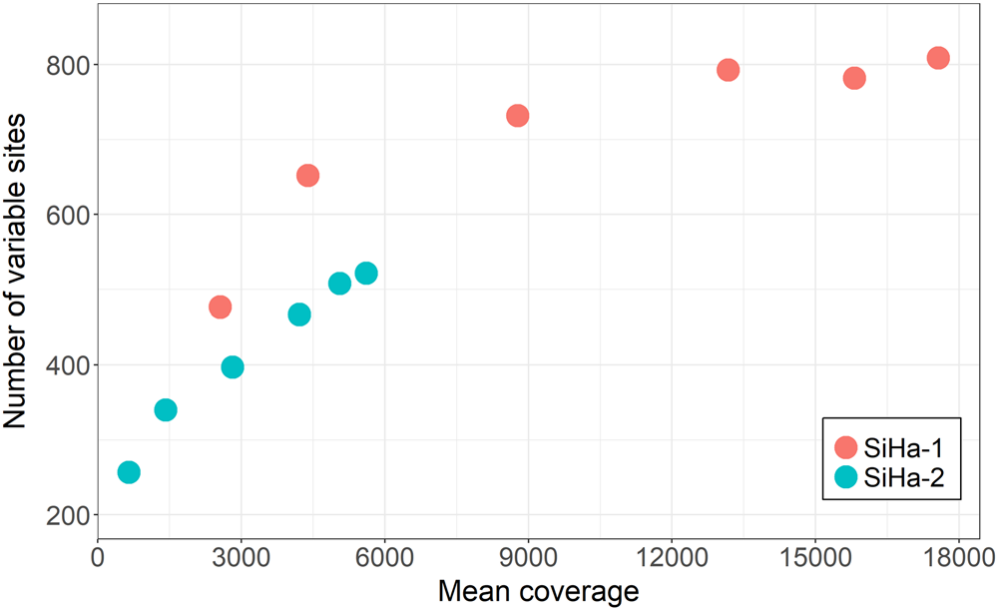
Number of variable sites in SiHa replicates. SiHa-1 (red dots) and SiHa-2 (blue dots) served as technical replicates to assess the variant calling performance. In SiHa libraries, sequenced on MiSeq and HiSeq 2500 platforms, increasing number of variable sites were detected with higher mean coverage.

Sequencing libraries of the SiHa cell line served as technical replicates to assess the variant calling performance. In both SiHa-1 and SiHa-2, more variable sites were detected with higher mean coverage (Fig. 4). Number of variable sites in SiHa-1 ranged from 477 to 809 and mean coverage ranged from 2554 to 17561. Number of variable sites in SiHa-2 ranged from 257 to 522 and mean coverage ranged from 646 to 5609 (Fig. 4; Supplementary Table S4). First, reproducibility of variant calling was assessed within the same SiHa sequencing library. Concordance rate of variable sites was calculated using HiSeq 2500 result as the reference value. The concordance rates varied from 92% (HiSeq downsampled 90%) to 45% (MiSeq) in SiHa-1 and from 89% (HiSeq downsampled 90%) to 27% (MiSeq) in SiHa-2 (Supplementary Table S4). Concordance rates of variants, including low frequency variation, between replicates (different library, same sequencing platform) were calculated to evaluate the effect of library preparation steps on the number of variable sites found in each sample. Concordance rates were 21% and 19% in SiHa-1 and SiHa-2, respectively (Supplementary Table S5).

### HPV genomic variability

**Figure 5:**
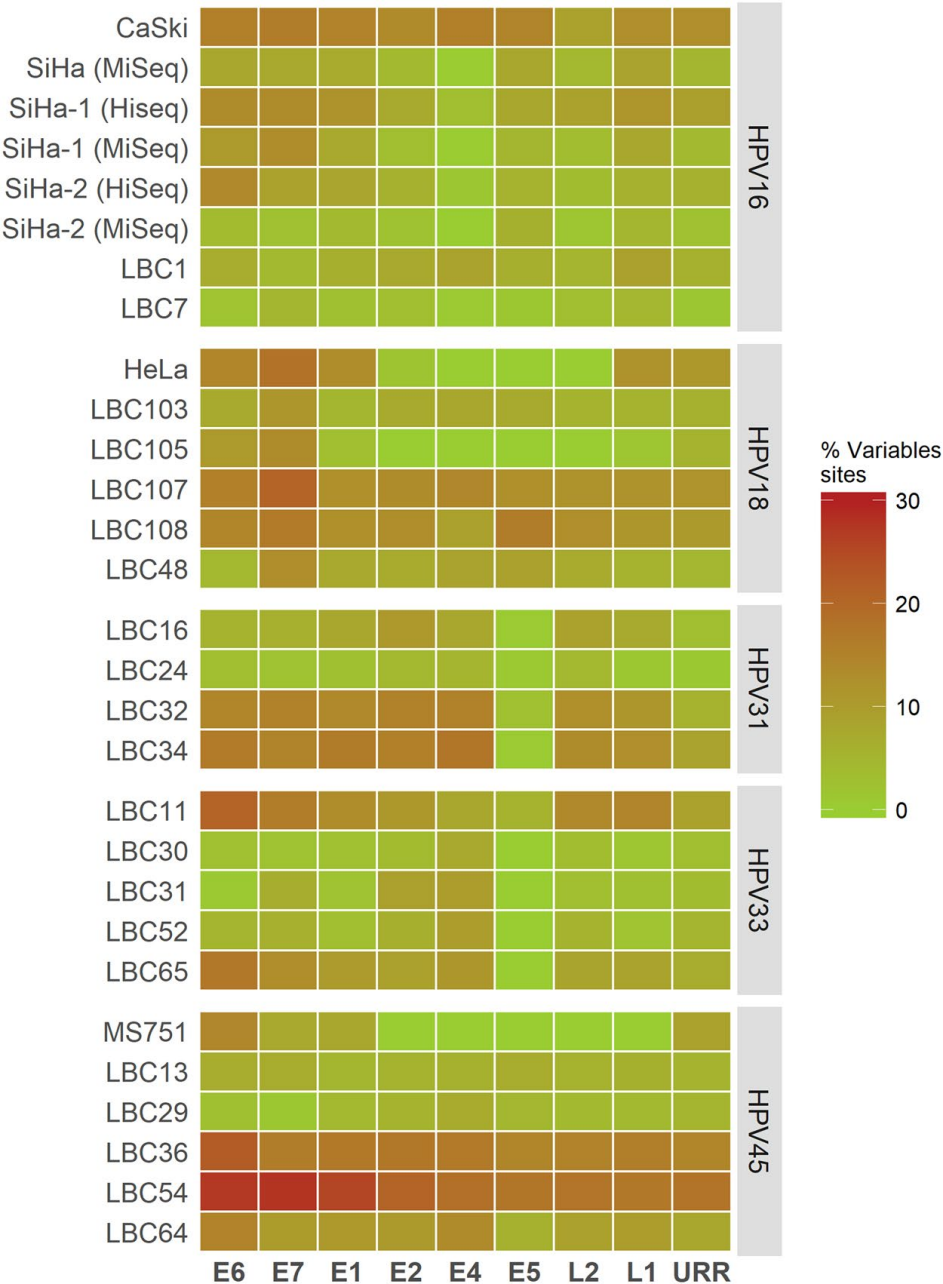
Proportion of variable sites in HPV genes in HPV positive samples. The number of variable sites was normalised by the length of each HPV gene. Gradient green (0% variable sites) to red (30% variable sites) color-coding of the results is shown to present the considerable variability in the samples throughout the HPV genome.

**Figure 6:**
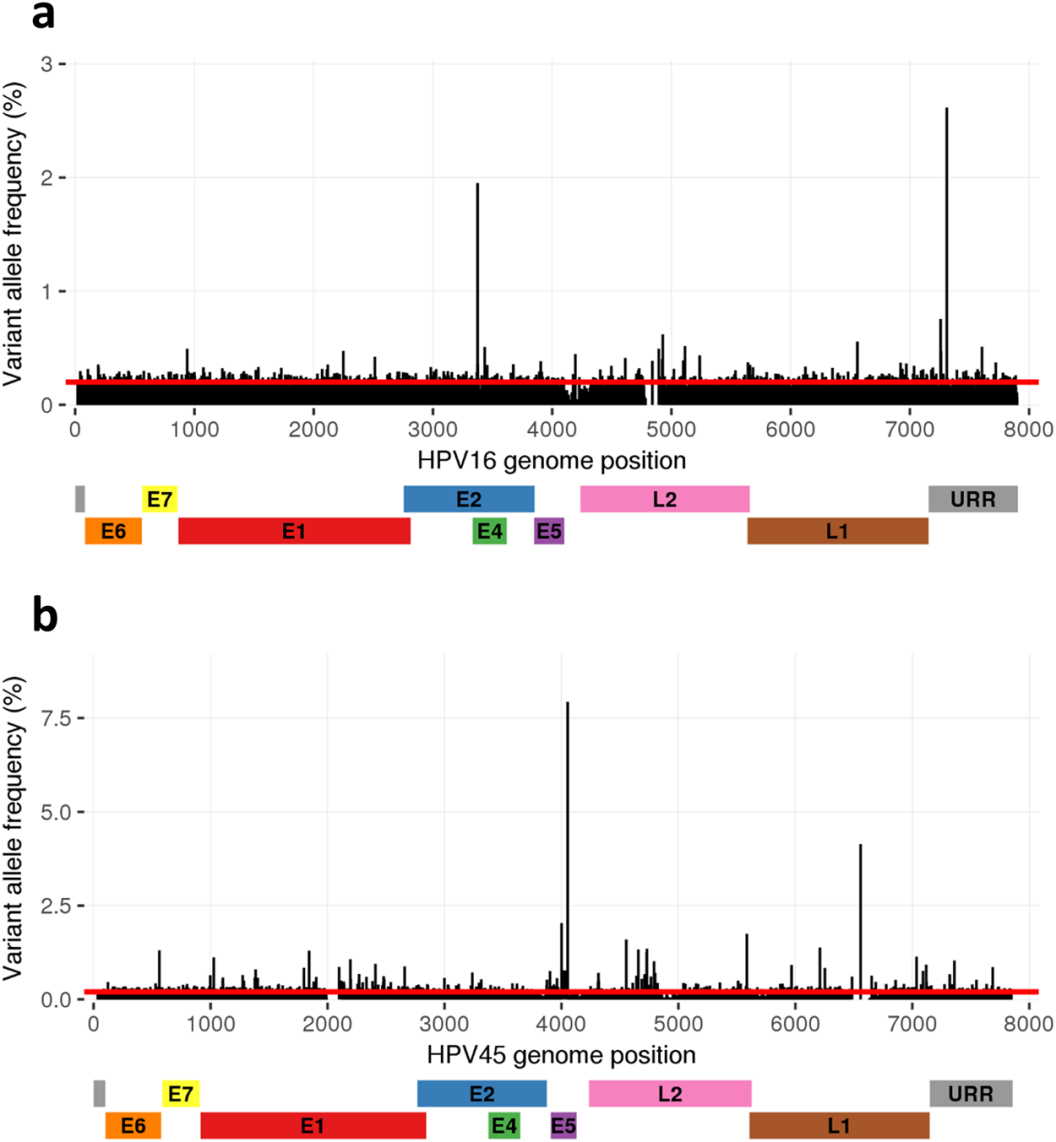
HPV nucleotide variation observed in two samples. The plots showing variable sites and variant allele frequency (%) in (**a**) CaSki, and (**b**) LBC54 are aligned to the respective target HPV genomes. The location of genes and URR is indicated below the genomic positions. The red line indicates the variant calling threshold value of 0.2%.

Variability was analysed in cell lines and LBC samples. Samples had variable sites (variant allele frequency >0.2% and coverage ≥100×) in all genes with the exception of regions that were deleted or had low sequencing coverage. The number of variable sites was normalised by the length of each HPV genomic region. Genomic regions had varying percentages of variable sites (0–28%) in each of the samples. Overall, there were samples within each HPV type that had >15% variable sites in at least one HPV gene (Fig. 5). Principally, samples with higher mean coverage had more variable sites (Supplementary Table S6), which is in line with the results from the variant analysis done on SiHa replicates (Fig. 4). CaSki had most variable sites (1017) of the cell lines and LBC54 had most variable sites (1641) of the clinical samples (Supplementary Table S6). A variant profile with variable site positions and variant allele frequency (VAF) is shown for CaSki and LBC54 (Fig. 6). Overall, the results show considerable variability in the samples throughout the HPV genome (Fig. 5, Supplementary Figs S6–S10).

## Discussion

Here, we present a novel cost-efficient approach, TaME-seq, for the simultaneous analysis of HPV variation and chromosomal integration. Previous methods have been less effective and/or limited to either one of the two analyses^29–34^. To demonstrate the performance of TaME-seq, we employed HPV16, 18, 31, 33 and 45 positive clinical samples, HPV positive cell lines and HPV plasmids. With 47% of the total of 154.8 million raw reads mapped on the target HPV reference genomes, TaME-seq proved to be highly efficient in HPV target enrichment. Other approaches for HPV target enrichment have reported much lower HPV mapping ratios^32,40^, requiring more sequencing and therefore at a higher sequencing cost. TaME-seq currently covers HPV16, 18, 31, 33 and 45, being the most common HPV genotypes in cervical cancer^5^. TaME-seq can be extended to cover additional HPV types, as well as other viruses, by implementing new primers to the method.

The ability of TaME-seq to detect chromosomal integration sites has been shown for the HPV positive cervical cancer cell lines CaSki, SiHa, HeLa and MS751. CaSki cells contain a high copy number (~600 copies/cell) of integrated full-length HPV16 arranged in concatemers^41,42^. SiHa (1–2 HPV16 copies/cell)^39,41^ and HeLa (10–50 HPV18 copies/cell)^43^ cells harbour integrated HPV genomes. MS751 cells contains integrated HPV45^35^, but in contrast to the product specification sheet (ATCC, Manassas, VA) no HPV18, which was verified in our analyses. For CaSki, 16 previously reported integration sites^30,32,37^ were detected by our method. In addition, three novel integration sites were identified. Known integration sites in SiHa^31,37,39^, HeLa^31,36^ and MS751^35^, as well as large deletions demonstrated in HeLa^36^ and MS751^35^, were confirmed by the TaME-seq method. Of the 21 LBC samples, HPV integration sites could only be detected in one sample, being in line with previous studies reporting no or few HPV integration events in LSIL/ASC-US samples^44,45^. However, other studies report integration events also in LSIL samples^32,46^. The detection of integrated forms of the virus is also dependent on the amount of episomes in the sample; low copy integration sites may remain undetected against a high background of episomal HPV.

The high sequencing coverage throughout the HPV genome enables detection of low frequency variants. Variant calling was evaluated using SiHa replicates to set the variant calling threshold. Previous studies have used variant calling thresholds of 0.5% or 1%^17,34^. With the high coverage provided by the TaME-seq method there is potential for detecting very low frequency variation. We have therefore analysed the variation using 0.2% as the variant calling threshold. Multiple and stringent filtering steps was included to filter out non-reliable variants, as we are approaching the inherent error rate profile of the PCR amplification and Illumina sequencing^47^. However, the threshold for variant calling is dependent on experimental and analytical basis and must be set according to the study aims.

The results from the SiHa analysis indicate that calling ultra-low frequency variants is dependent on the sequencing coverage. Lower sequencing coverage results in the detection of fewer variants and less concordance between sample replicates. In order to find ultra-low frequency variants, high sequencing coverage is required. Figure 4 shows that at the mean coverage of 12000×, the number of variants in SiHa-1 is approaching saturation. This indicates that more variants are not likely to be found even with higher sequencing coverage. Finally, differences in sequencing coverage affect the number of variable sites found, but also experimental approaches due to stochastic sampling and variant calling can fail to reveal low frequency variants. Overall, our results uncover low frequency variants in the samples, potentially introduced by DNA repair mechanisms and APOBEC enzyme mediated DNA editing^48–50^, although some bias may be introduced by PCR and sequencing. Variable sites are present in all genes of the studied HPV types. Traditionally, studies have focused on sequence variation on a viral sublineage level^13–16^ or the high variability has been interpreted as HPV variant co-infections^29^. The development of NGS technologies has provided comprehensive tools for the study of HPV genomic variability. Recent studies have reported high HPV variability that may be evidence of intra-host viral evolution and adaptation generated during a chronic HPV infection^17–20^.

Our study has some limitations. Firstly, TaME-seq is not intended for determining HPV genotypes and we recommend it for analyses of HPV variability and integration events in samples with known HPV status. Secondly, due to variation in amplification efficacy, an uneven coverage is seen for different genomic regions. Sudden drops in the coverage, that are not genomic deletions, may be due to suboptimal primer performance or poor alignment against the reference genomes. This issue can be solved partly by designing new primers covering these regions and optimising the primer performance. Also, the read alignment step can be further optimised. Alternatively, alignment could be performed by *de novo* assembly to create consensus sequences for the alignment. Thirdly, enough viral DNA and good dsDNA quality are important for achieving consistent tagmentation results in the Nextera protocol^51^. Sample preparation of the excluded LBC samples failed likely due to very low viral load in the samples, which was not quantified separately.

In summary, we have developed a NGS approach that allows the simultaneous study of HPV genomic variability and chromosomal integration. TaME-seq is applicable to large sample cohorts due to its highly efficient target enrichment, leading to less off-target sequences and therefore reduced sequencing cost. Comprehensive studies on HPV intra-host variability generated during a persistent infection will improve our understanding of viral carcinogenesis. Efficient identification of HPV genomic variability and integration sites will be important both for the study of HPV evolution, adaptability and may be a useful tool for cervical cancer diagnostics.

## Methods

### Samples

Anonymised LBC samples from routine cervical cancer screening were included in the study, comprising cases of atypical squamous cells of undetermined significance (ASC-US) and low-grade squamous intraepithelial lesions (LSIL). HPV positive samples with the cobas 4800 HPV test (Roche Molecular Diagnostics, Pleasanton, CA) were extracted for DNA using the automated system NucliSENS easyMAG (BioMerieux Inc., France) with off-board lysis. The samples were HPV genotyped using the modified GP5+/6+ PCR protocol (MGP)^52^, followed by HPV type-specific hybridisation using Luminex suspension array technology^53^ or the Anyplex™ II HPV28 assay (Seegene, Inc., Seoul, Korea). LBC samples (n = 31) were positive for HPV16, 18, 31, 33 or 45 alone, or had multiple infections including at least one of the five types. DNA extracted from the HPV positive cervical carcinoma cell lines CaSki, SiHa, HeLa and MS751 (ATCC, Manassas, VA) served as positive controls. WHO international standards for HPV 16 (1st WHO International Standard for Human Papillomavirus Type 16 DNA, NIBSC code: 06/202) and 18 (1st WHO International Standard for Human Papillomavirus Type 18 DNA, NIBSC code: 06/206)(NIBSC, Potters Bar, Hertfordshire, UK) and a plasmid containing the strain HPV33^54^ were used as additional positive controls. Laboratory-grade water and DNA from an HPV negative human sample were included as negative controls. DNA was quantified by the fluorescence-based Qubit dsDNA HS assay (Thermo Fisher Scientific Inc.,Waltham, MA, USA).

### Primer design

HPV16, 18, 31, 33, and 45 whole genome reference and variant sequences were obtained from the PapillomaVirus Episteme (PaVE) database^55^. All the available reference and variant sequences within an HPV type were aligned using the multiple sequence alignment tool ClustalO^56^. The sequence alignment was converted to a consensus sequence for each HPV type in CLC Sequence viewer version 7.7.1 (QIAGEN Aarhus A/S). TaME-seq HPV primers were designed using Primer3^57^ and HPV consensus sequences as the source sequence. Finally, primers were modified by adding an Illumina TruSeq-compatible adapter tail (5′-AGACGTGTGCTCTTCCGATCT-3′) to the 5′-end and then synthesised by Thermo Fisher Scientific, Inc. (Waltham, MA).

### Library preparation and sequencing

Primer pools for each HPV type were prepared by combining primers separately in equal volumes. Samples were subjected to tagmentation using Nextera DNA library prep kit (Illumina, Inc., San Diego, CA). Tagmented DNA was purified using DNA Clean & Concentrator™-5 columns (Zymo Research, Irvine, CA) according the manufacturer’s instructions or ZR-96 DNA Clean & Concentrator™-5 plates (Zymo Research, Irvine, CA) according to the Nextera® DNA Library Prep Reference Guide (15027987 v01) before PCR amplification for target enrichment. Amplification was performed using Qiagen Multiplex PCR Master mix (Qiagen, Hilden, Germany) according to the manufacturer’s instructions. For each sample, two PCR reactions were performed separately with 0.75 µM of HPV primer pools, 0.5 µM of i7 index primers (adapted from Kozich *et al*.^58^) and 1 µl of i5 index primers from the Nextera index kit (Illumina, Inc., San Diego, CA). The cycling conditions were as follows: initial denaturation and hot start at 95 °C for 5 minutes; 30 cycles at 95 °C for 30 seconds, at 58 °C for 90 seconds and at 72 °C for 20 seconds; final extension at 68 °C for 10 minutes. Following amplification, libraries were pooled in equal volumes and the final sample pool was purified with Agencourt^®^ AMPure^®^ XP beads (Beckman Coulter, Brea, CA). The quality and quantity of the pooled libraries were assessed on Agilent 2100 Bioanalyzer using Agilent High Sensitivity DNA Kit (Agilent Technologies Inc., Santa Clara, CA) and by qPCR using KAPA DNA library quantification kit (Kapa Biosystems, Wilmington, MA). Sequencing was performed on the MiSeq platform (Illumina, Inc., San Diego, CA) or on the HiSeq 2500 platform (Illumina, Inc., San Diego, CA). Samples were sequenced as 151 bp paired-end reads and two 8 bp index reads.

### Sequence alignment

Raw paired-end reads were trimmed for adapters, HPV primers, quality (-q 20) and finally for minimum length (-m 50) using cutadapt (v1.10)^59^. Trimmed reads were mapped to human (GRCh38/hg38) and HPV16, 18, 31, 33 and 45 reference genomes obtained from the PaVE database^55^ using HISAT2 (v2.1.0)^60^. Mapping statistics and sequencing coverage were calculated using the Pysam package^61^ with an in-house Python (v3.5.4) script. Downstream analysis was performed using an in-house R (v3.4.4) script. Results from both reactions of the same sample were combined and method performance was then evaluated based on the percentage of obtained reads mapped to the HPV reference genome, mean sequencing coverage and percentage of HPV reference genome coverage for each sample. Further analysis was performed when a sample had >20000 reads mapped to the target HPV reference genome. The target HPV genomes correspond to the HPV types for which the samples were reported positive by HPV genotyping.

### Detecting HPV-human integration sites

The paired-end reads that mapped (HISAT2) with one end to a human chromosome and the other end to the target HPV reference genome were identified as discordant read pairs. If a specific position had ≥2 read pairs with unique start or end coordinates, it was considered as a potential integration site. To determine the exact position of HPV-human integration breakpoints, previously unmapped reads were remapped to human and HPV reference genomes (as above) using the LAST (v876) aligner (options -M -C2)^62^. Positions covered by ≥3 junction reads, with unique start or end coordinates, were considered as potential integration breakpoints. Integration site detection was not based on reads sharing the same start and end coordinates as these reads were considered as potential PCR duplicates. Selected HPV integration breakpoints were confirmed by PCR amplification and Sanger sequencing.

### Sequence variation analysis

Mapped nucleotide counts over HPV reference genomes and average mapping quality values of each nucleotide were retrieved from BAM files and variant calling was performed using an in-house R script. To reduce the effects of PCR amplification and sequencing artefacts in the variation analysis, filtering was applied before the variant calling. Nucleotides seen ≤2 times in each position and nucleotides with mean Phred quality score of <20 were filtered out. Nucleotide counts from both reactions of the same sample were combined and variant allele frequencies (VAF) of the three minor alleles in each position were calculated. If results from either of the reaction showed >5 times larger VAF with <20% of the total coverage, it was discarded from variant calling. Finally, variants were called if VAF was >0.2% and coverage was ≥100×.

Two sequencing libraries of SiHa cell line served as technical replicates to assess the variant calling performance. The technical replicates were sequenced on the MiSeq platform or on the HiSeq 2500 platform. In addition, HiSeq raw sequencing data was downsampled randomly and defined portions (90%, 75%, 50% and 25%) of the original reads were further analysed. Reproducibility of calling variants in the replicates was assessed by calculating concordance rate. The concordance rate (R_c_) between duplicates was defined as follows:

$${R}_{c}=\frac{{N}_{c}}{mean({N}_{1},{N}_{2})}$$

where N_c_ was the number of concordant variants between a pair of replicate samples, and N_1_ and N_2_ were the total number of variants detected in each of the duplicated sample.

### Ethical approval

This study was approved by the regional committee for medical and health research ethics, Oslo, Norway [2017/447] and we confirm that all experiments were performed in accordance with the committee’s guidelines and regulations.

## Electronic supplementary material

Supplementary information

## Data Availability

Sequence data from cell lines will be available at European Nucleotide Archive (ENA) accession number ERP111061. Plasmids are third party property and requests must be made to International Human Papillomavirus Reference Center and Institut Pasteur. Sequencing data from clinical samples will be available from the authors upon request with obtained ethical approval. Clinical sequence data may be deposited at the European Genome-phenome Archive (EGA) (ethical and legal assessments are on-going).
