# Supplementary material for: TaME-seq: An efficient sequencing approach for characterisation of HPV genomic variability and chromosomal integration
Source: Sci Rep. 2019 Jan 24;9:524. doi: 10.1038/s41598-018-36669-6 (PMC6345795; doi:10.1038/s41598-018-36669-6)
Supplement: Supplementary file 1 — Supplementary information [file 41598_2018_36669_MOESM1_ESM.docx]

**TaME-seq: An efficient sequencing approach for characterisation of HPV genomic variability and chromosomal integration**

Sonja Lagström^1,2^, Sinan Uğur Umu^2^, Maija Lepistö^3^, Pekka Ellonen^3^, Roger Meisal^1^, Irene Kraus Christiansen^1,4^, Ole Herman Ambur^5^, Trine B. Rounge^2,^*

Author affiliations:

^1^Department of Microbiology and Infection Control, Akershus University Hospital, Lørenskog, Norway

^2^Department of Research, Cancer Registry of Norway, Oslo, Norway

^3^Institute for Molecular Medicine Finland, University of Helsinki, Helsinki, Finland

^4^Department of Clinical Molecular Biology (EpiGen), Division of Medicine, Akershus University Hospital and University of Oslo, Lørenskog, Norway

^5^Faculty of Health Sciences, OsloMet - Oslo Metropolitan University, Oslo, Norway

*Corresponding author:

E-mail: trine.rounge@kreftregisteret.no

**Supplementary Table S1.** Read counts and sequencing coverage of HPV positive samples that were excluded from the analysis.

| **Sample** | **Sample type** | **Raw reads** | **Trimmed reads** | **Reads mapped to target HPV** | **% Reads mapped to target HPV** | **Mean coverage** | **Fraction of genome covered by minimum** | |
| --- | --- | --- | --- | --- | --- | --- | --- | --- |
|  |  |  |  |  |  |  | **10×** | **100×** |
| ***HPV16*** |  |  |  |  |  |  |  |  |
| LBC43^a^ | LBC | 47788^c^ | 34778 | 15283 | 32% | 205 | 0.86 | 0.44 |
| ***HPV18*** |  |  |  |  |  |  |  |  |
| MS751^a^ | Cell line | 890142^b^ | 837366 | 220 | 0.0% | 3 | 0.08 | 0.00 |
| LBC110^a^ | LBC | 1673786^b^ | 1464308 | 7958 | 0.5% | 118 | 0.82 | 0.36 |
| LBC10^a^ | LBC | 144822^b^ | 107538 | 1133 | 0.8% | 17 | 0.28 | 0.04 |
| LBC18^a^ | LBC | 700120^b^ | 107538 | 160 | 0.0% | 2 | 0.05 | 0.00 |
| LBC41^a^ | LBC | 2839890^c^ | 1996944 | 16982 | 0.6% | 212 | 0.62 | 0.40 |
| LBC56 | LBC | 508874^b^ | 406150 | 647 | 0.1% | 9 | 0.32 | 0.00 |
| ***HPV31*** |  |  |  |  |  |  |  |  |
| LBC8 | LBC | 120100^b^ | 84434 | 3220 | 0.4% | 47 | 0.60 | 0.14 |
| LBC17^a^ | LBC | 330244^b^ | 228388 | 712 | 0.2% | 10 | 0.18 | 0.03 |
| LBC18^a^ | LBC | 214800^b^ | 163400 | 439 | 0.2% | 6 | 0.17 | 0.00 |
| ***HPV45*** |  |  |  |  |  |  |  |  |
| LBC40 | LBC | 205342^b^ | 166784 | 107 | 0.1% | 2 | 0.05 | 0.00 |

^a^ Sample has multiple HPV infections.

^b^ Sequenced on MiSeq sequencing platform.

^c^ Sequenced on HiSeq 2500 sequencing platform.

**Supplementary Table S2.** Read counts and sequencing coverage of HPV negative control samples.

| **Sample** | **Raw reads** | **Trimmed reads** | **Reads mapped to target HPV** | **% Reads mapped to target HPV** | **Mean coverage** | **Fraction of genome covered by minimum** | | |
| --- | --- | --- | --- | --- | --- | --- | --- | --- |
|  |  |  |  |  |  | **10×** | **100×** | |
| ***HPV16*** |  |  |  |  |  |  |  |  |
| H_2_O | 1060^b^ | 794 | 482 | 45% | 7 | 0.24 | 0.00 | |
| Human | 38710^b^ | 33928 | 54 | 0.1% | 1 | 0.00 | 0.00 | |
| ***HPV18*** |  |  |  |  |  |  |  | |
| H_2_O | 214^b^ | 146 | 0 | 0.0% | 0 | 0.00 | 0.00 | |
| Human | 496112^b^ | 412056 | 51 | 0.0% | 1 | 0.00 | 0.00 | |
| ***HPV31*** |  |  |  |  |  |  |  |  |
| H_2_O | 810^b^ | 594 | 0 | 0.0% | 0 | 0.00 | 0.00 | |
| Human | 340858^b^ | 285822 | 0 | 0.0% | 0 | 0.00 | 0.00 | |
| ***HPV33*** |  |  |  |  |  |  |  |  |
| H_2_O | 4828^b^ | 3406 | 297 | 6.2% | 4 | 0.14 | 0.00 | |
| Human | 3010522^c^ | 1707226 | 22 | 0.0% | 0 | 0.00 | 0.00 | |
| ***HPV45*** |  |  |  |  |  |  |  |  |
| H_2_O | 178^b^ | 144 | 16 | 9.0% | 0 | 0.00 | 0.00 | |
| Human | 1237502^b^ | 1075344 | 72 | 0.0% | 1 | 0.01 | 0.00 | |

^b^ Sequenced on MiSeq sequencing platform.

^c^ Sequenced on HiSeq 2500 sequencing platform.

**Supplementary Table S3.** Integration breakpoints confirmed by PCR amplification and Sanger sequencing.

| **Sample** | **Junction sequence^a^** | **HPV** | | | |  | **Human** | | |
| --- | --- | --- | --- | --- | --- | --- | --- | --- | --- |
|  |  | **Type** | **Start** | **End** | **ORF** |  | **Chromosomal locus** | **Start** | **End** |
| CaSki^b^ | GCCAAATATATATATATATACACACACACATATATATGTATACTATATACTATAGTATATACAGTATATATAGTATATATGTAAACTATAGCCAAATATATATATAGCCATTAGTTGCAGTTCAATTGCTTGTAATGCTTTATTCTTTGATACAGCCAGCGTTGGCACCACCTGGTGGTTAATATGTTTAAATCCCATTTCTCTGGCCTTGTAATAAATAGCACATTCTAGGCGCATGTGTTTCCAATAG | HPV16 | 2987 | 2848 | E2 |  | Xq27.3 | 145708341 | 145708231 |
| CaSki | TAATATAAGGGGTCGGTGGACCGGTCGATGTATGTCTTGTTGCAGATCATCAAGAACACGTAAAGAAACCCAGCTGTAATCATGCATGGAGATACACCTACATTGCATGAATATATGTGCCACATTTTCTTAATCCAGTCTATCATTGTTGGACATTTGGGTTGGTTCC | HPV16 | 481 | 598 | E6 |  | 20p11.1 | 26341312 | 26341369 |
| CaSki | TTCCCTTTCAGAGAGCACGTTTAAAACACCCTTTTTGTAGTATCTGGAAGTGCACATTTGGAGGGCTTTGATGCATATGGTGAAAAAGGAAATGACTCATATGATACAACTGCTAAACGCAAAAAACGTAAGCTGTAAGTATTGTATGTATGTTGAATTAGTGTTGTTTGTTGTTTATATGTTTGTATGTGCTTGTATGTGCT | HPV16 | 7123 | 7221 | L1 |  | 20p11.1 | 26357830 | 26357922 |
| LBC105 | TACAAGTGACAATAGCAATATAGAAAATGTAAATCCACAATGTACCATAGCACAATTAAAAGACTTGTTAAAAGTAAACAATAAACAAGGAGCTATGTTAGCATGCCACCATGCCACGCCCAGTTAATTTTTGTATTTTTGTAGAGACGGGGTTTCACCATGTTGGCCAGGCTGGTCTCGAACTCTCGAGCTCAAGTGATTCACCTGCCTCGGCCTCCCAAAGTGCA | HPV18 | 1459 | 1561 | E1 |  | 7q11.23 | 74525628 | 74525503 |
| LBC105 | CAAGCCTGGGGCTATTTCTAGGCGAGAGGTGGCAGTGACTTGAGCCAGGGCAGGGACAGTAGGGGTGGAGGTTTGGAAAACGGCTGGAGCAGAACTTCTGTGTCACTGTGAGGTACCATTGGATATTTGTCAGTCTATTTGTAAATATCCTGATTATTTACAAATGTCTGCAGATCCTTATGGGGATTCCATGTTTTTTTGCTTACGGCGTGAGCAGCTTTTTGCTAGGCATTTTTGGAATAGGGCAGGT | HPV18 | 6264 | 6407 | L1 |  | 7q11.23 | 74515867 | 74515764 |

^a^ Black letters: human sequence (aligned to GRCh38/hg38); red letters: HPV sequence (aligned to HPV reference sequences obtained from the PaVE database); green letters: nucleotides shared between HPV and human genomes; blue letters: nucleotides that did not align.

^b^ Previously reported integration site that was used as a control.

**Supplementary Table S4.** Reproducibility of variant calling was assessed within same SiHa sequencing libraries. Concordance rate of variable sites was calculated using HiSeq 2500 results as reference.

| **Sample** | **Sequence platform (downsampled)** | **Mean coverage** | **Variable sites** | **% Concordance** |
| --- | --- | --- | --- | --- |
| SiHa-1 | HiSeq 2500 | 17561 | 809 | - |
|  | HiSeq 2500 (90%) | 15809 | 782 | 92 |
|  | HiSeq 2500 (75%) | 13176 | 793 | 84 |
|  | HiSeq 2500 (50%) | 8773 | 732 | 73 |
|  | HiSeq 2500 (25%) | 4386 | 652 | 61 |
|  | MiSeq | 2554 | 477 | 45 |
| SiHa-2 | HiSeq 2500 | 5609 | 522 | - |
|  | HiSeq 2500 (90%) | 5057 | 508 | 89 |
|  | HiSeq 2500 (75%) | 4212 | 467 | 78 |
|  | HiSeq 2500 (50%) | 2811 | 397 | 64 |
|  | HiSeq 2500 (25%) | 1415 | 340 | 48 |
|  | MiSeq | 646 | 257 | 27 |

**Supplementary Table S5.** Concordance rate of variable sites in two technical replicates of SiHa sequenced on a same platform.

| **Sample** | **Sequence platform** | **Mean coverage** | **Variable sites** | **% Concordance** |
| --- | --- | --- | --- | --- |
| SiHa-1 | HiSeq 2500 | 17561 | 809 | 21 |
| SiHa-2 |  | 5609 | 522 |  |
| SiHa-1 | MiSeq | 2554 | 477 | 19 |
| SiHa-2 |  | 646 | 257 |  |

**Supplementary Table S6.** Mean coverage, total number of variable sites and percentage of variable sites in each HPV genes in the HPV positive cell lines and LBC samples.

| **Sample** | **Mean coverage** | **Variable sites** | **% Variable sites** | | | | | | | | |
| --- | --- | --- | --- | --- | --- | --- | --- | --- | --- | --- | --- |
|  |  |  | **E6** | **E7** | **E1** | **E2** | **E4** | **E5** | **L2** | **L1** | **URR** |
| ***HPV16*** |  |  |  |  |  |  |  |  |  |  |  |
| CaSki | 184716 | 1017 | 15.7 | 16.2 | 15.1 | 13.6 | 15.6 | 14.7 | 8.9 | 12.8 | 12.7 |
| SiHa^b^ | 1018 | 500 | 7.8 | 7.4 | 7.1 | 4.0 | 0.3 | 7.9 | 4.4 | 8.6 | 4.8 |
| SiHa-1^c^ | 17561 | 809 | 13.4 | 13.5 | 12.0 | 7.3 | 3.1 | 7.9 | 8.8 | 11.5 | 9.4 |
| SiHa-1^b^ | 2554 | 477 | 10.5 | 13.1 | 7.5 | 3.2 | 0.3 | 4.8 | 3.3 | 7.6 | 4.0 |
| SiHa-2^c^ | 5609 | 522 | 13.8 | 8.8 | 8.3 | 5.8 | 1.4 | 5.6 | 3.6 | 5.9 | 5.9 |
| SiHa-2^b^ | 646 | 257 | 3.8 | 2.4 | 3.9 | 2.3 | 0.0 | 6 | 1.6 | 4.7 | 2.5 |
| LBC1 | 1124 | 525 | 6.7 | 4.0 | 6.2 | 7.5 | 8.7 | 6.3 | 5.0 | 9.1 | 5.8 |
| LBC7 | 384 | 244 | 1.9 | 4.7 | 2.7 | 3.0 | 0.7 | 1.2 | 3.1 | 4.5 | 1.4 |
| ***HPV18*** |  |  |  |  |  |  |  |  |  |  |  |
| HeLa | 5897 | 679 | 14.5 | 18.2 | 13.1 | 1.8 | 0.0 | 0.0 | 0.0 | 12.1 | 11.0 |
| LBC103 | 1056 | 456 | 7.3 | 11.3 | 4.8 | 7.2 | 7.9 | 7.2 | 5.2 | 5.4 | 5.7 |
| LBC105 | 484 | 222 | 10.5 | 13.5 | 3.0 | 0.0 | 0.0 | 0.0 | 0.0 | 1.6 | 5.3 |
| LBC107 | 14663 | 999 | 15.5 | 20.8 | 12.6 | 13.3 | 14.2 | 12.6 | 12.0 | 11.9 | 11.8 |
| LBC108 | 46691 | 967 | 14.5 | 16.7 | 12.5 | 13.0 | 9.0 | 16.2 | 12.7 | 11.2 | 10.4 |
| LBC48 | 988 | 521 | 4.4 | 12.9 | 7.5 | 7.0 | 8.6 | 9.0 | 7.0 | 5.6 | 4.6 |
| ***HPV31*** |  |  |  |  |  |  |  |  |  |  |  |
| LBC16 | 1065 | 561 | 5.3 | 6.1 | 7.8 | 10.7 | 7.8 | 0.4 | 8.9 | 7.3 | 3.1 |
| LBC24 | 355 | 206 | 3.1 | 2.0 | 2.6 | 4.3 | 4.9 | 0.8 | 4.4 | 1.3 | 1.0 |
| LBC32 | 18983 | 932 | 14.4 | 15.2 | 13.8 | 15.2 | 15.2 | 2.4 | 12.5 | 11.2 | 5.5 |
| LBC34 | 23790 | 1060 | 16.7 | 14.8 | 16.6 | 15.4 | 17.5 | 0.4 | 13.4 | 12.8 | 8.6 |
| ***HPV33*** |  |  |  |  |  |  |  |  |  |  |  |
| LBC11 | 12038 | 1032 | 20.9 | 16.3 | 13.2 | 11.0 | 7.9 | 5.3 | 13.8 | 14.7 | 8.9 |
| LBC30 | 303 | 202 | 2.4 | 2.0 | 2.5 | 3.7 | 7.5 | 0.0 | 3.3 | 1.5 | 3.1 |
| LBC31 | 544 | 272 | 0.9 | 6.5 | 2.1 | 9.2 | 9.9 | 0.0 | 2.9 | 2.6 | 3.5 |
| LBC52 | 439 | 313 | 4.9 | 6.1 | 3.2 | 6.2 | 9.9 | 0.0 | 5.1 | 1.9 | 5.0 |
| LBC65 | 1993 | 732 | 17.1 | 12.9 | 10.3 | 9.0 | 11.1 | 0.0 | 8.4 | 8.7 | 6.9 |
| ***HPV45*** |  |  |  |  |  |  |  |  |  |  |  |
| MS751 | 845 | 321 | 14.3 | 7.5 | 7.9 | 0.0 | 0.0 | 0.0 | 0.0 | 0.1 | 8.9 |
| LBC13 | 849 | 432 | 6.7 | 6.9 | 4.7 | 5.4 | 5.9 | 6.8 | 5.5 | 6.0 | 5.2 |
| LBC29 | 614 | 329 | 2.7 | 1.2 | 4.4 | 5.2 | 7.0 | 4.5 | 3.9 | 4.2 | 4.9 |
| LBC36 | 22093 | 1279 | 22.2 | 16.2 | 17.0 | 17.1 | 16.8 | 14.4 | 15.0 | 16.0 | 14.4 |
| LBC54 | 256857 | 1641 | 27.5 | 28.0 | 25.8 | 20.8 | 18.7 | 17.6 | 17.7 | 17.2 | 18.0 |
| LBC64 | 3943 | 782 | 15.1 | 10.0 | 10.7 | 10.4 | 13.6 | 5.9 | 9.0 | 9.8 | 7.9 |

^b^ Sequenced on MiSeq sequencing platform.

^c^ Sequenced on HiSeq 2500 sequencing platform.

**a b**

**
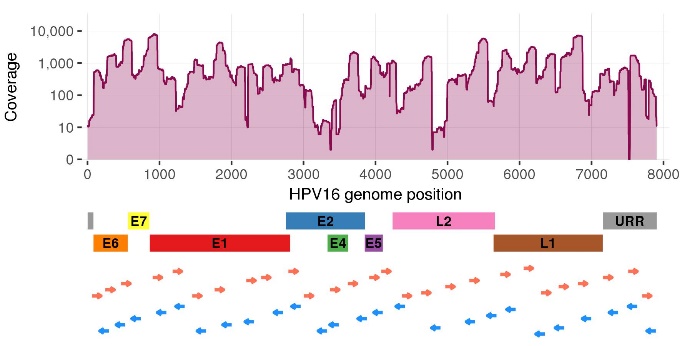

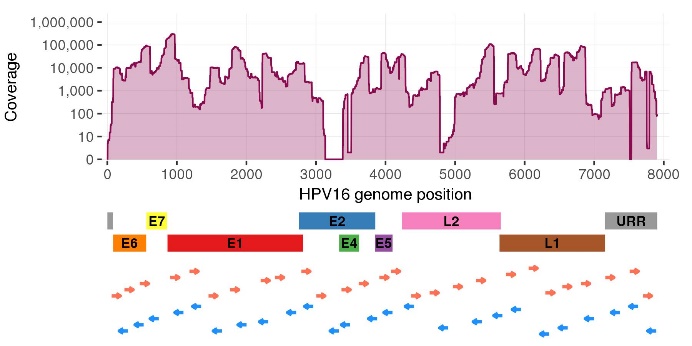
**

**c d**


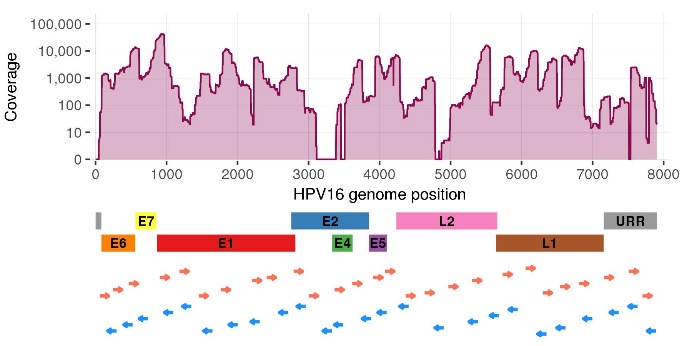

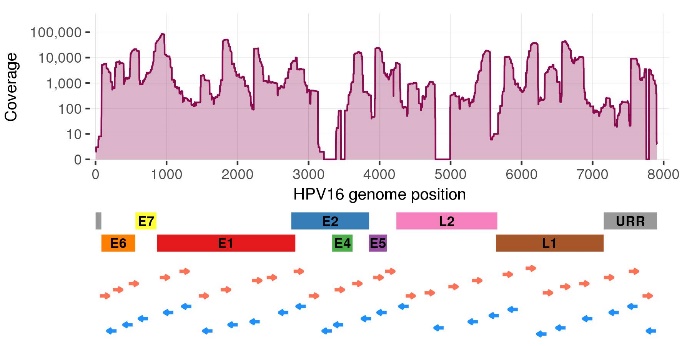


**e f**


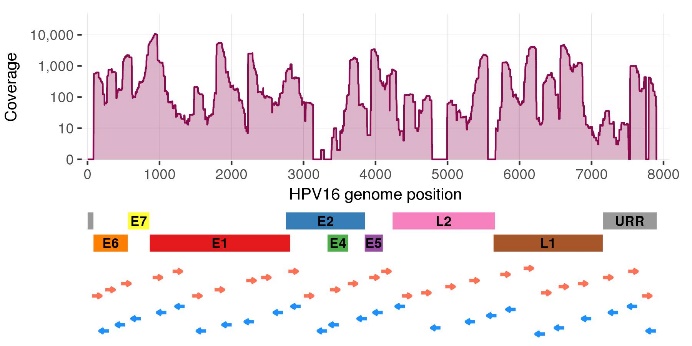

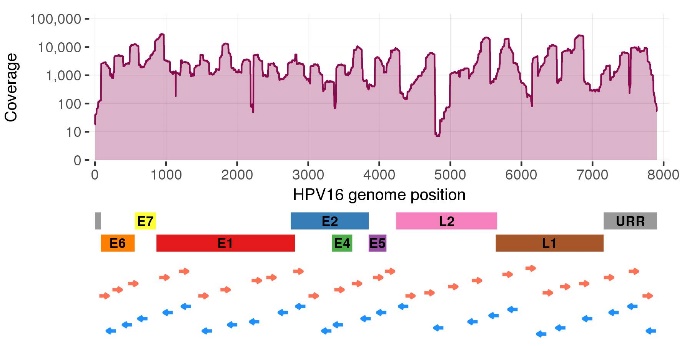


**g h**


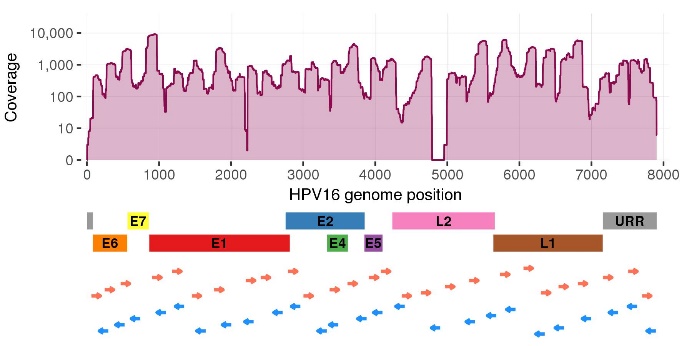

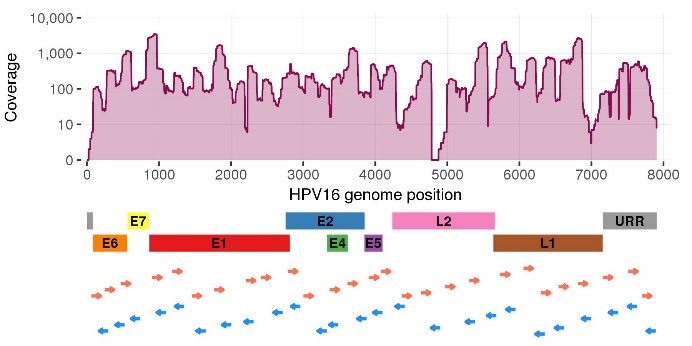


**Supplementary Figure S1.** HPV genome sequencing coverage of HPV16 positive samples a) SiHa (sequenced on MiSeq), b) SiHa-1 (sequenced on HiSeq), c) SiHa-1 (sequenced on MiSeq), d) SiHa-2 (sequenced on HiSeq), e) SiHa-2 (sequenced on MiSeq), f) WHO standard for HPV16, g) LBC1, and h) LBC7. The coverage plots are aligned to the HPV16 genome. The location of early (E1, E2, E4-7), late (L1, L2) genes, URR, and forward (red arrows) and reverse (blue arrows) HPV16 primers is indicated below the genomic positions.

**a b**

**
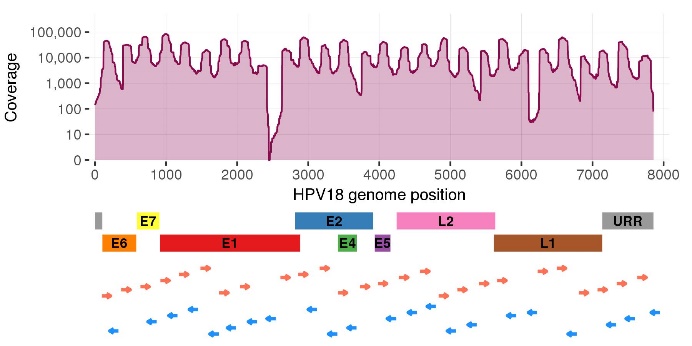

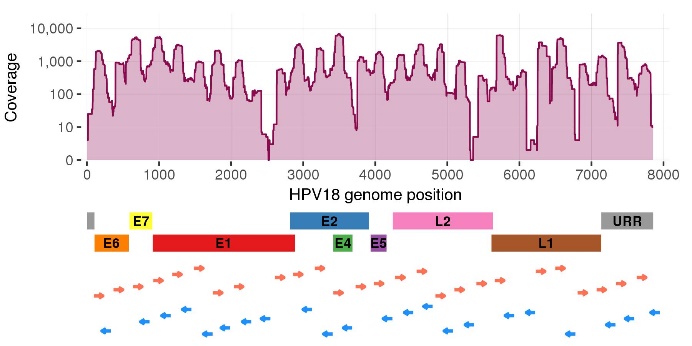
**

**c d**


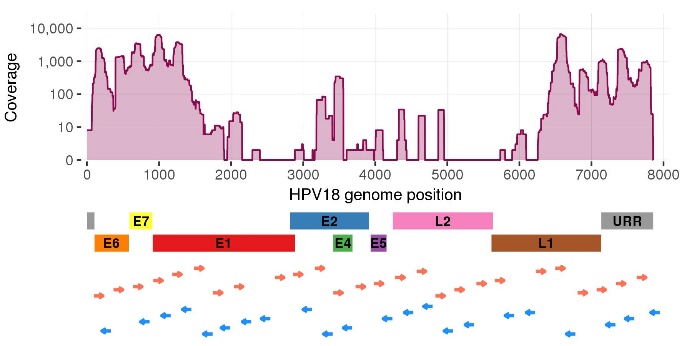

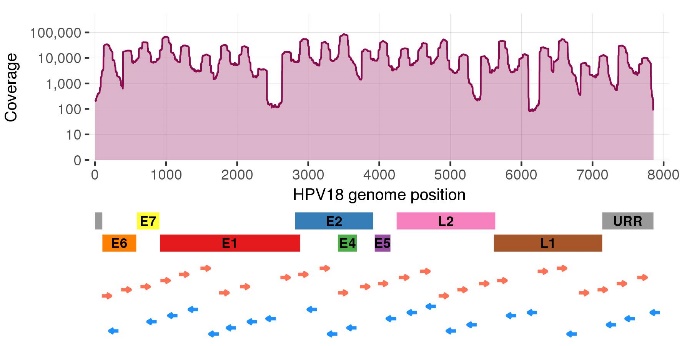


**e f**


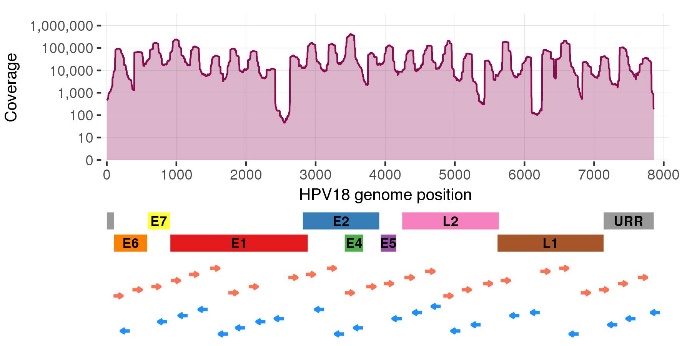

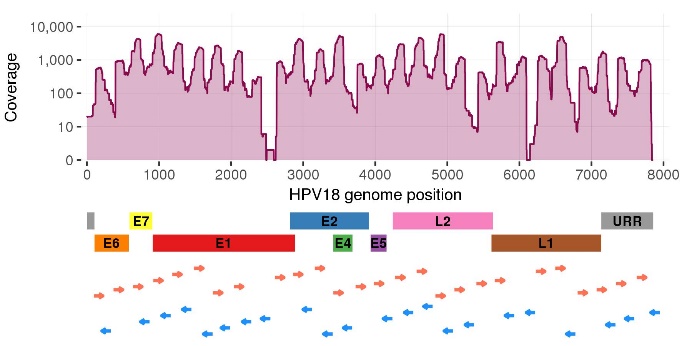


**Supplementary Figure S2.** HPV genome sequencing coverage of HPV18 positive samples a) WHO standard for HPV18, b) LBC103, c) LBC105, d) LBC107, e) LBC108, and f) LBC48. The coverage plots are aligned to the HPV18 genome. The location of early (E1, E2, E4-7), late (L1, L2) genes, URR, and forward (red arrows) and reverse (blue arrows) HPV18 primers is indicated below the genomic positions.

**a b**

**
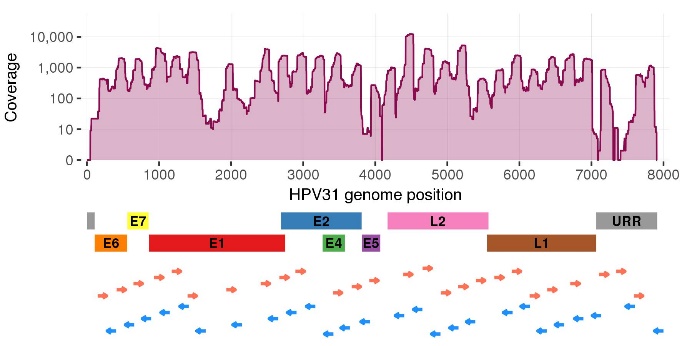

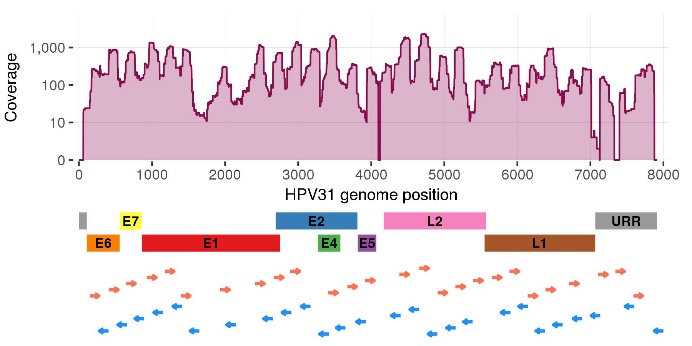
**

**c**


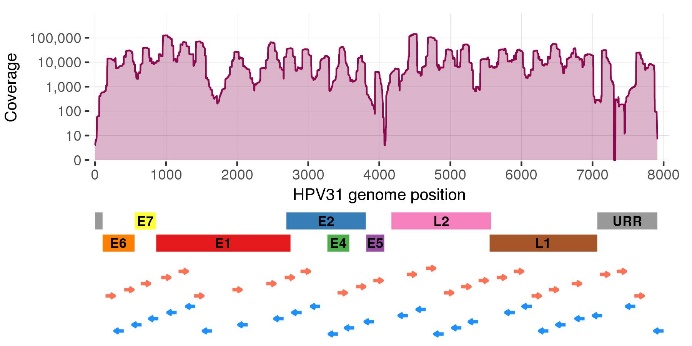


**Supplementary Figure S3.** HPV genome sequencing coverage of HPV31 positive samples a) LBC16, b) LBC24, and c) LBC32. The coverage plots are aligned to the HPV31 genome. The location of early (E1, E2, E4-7), late (L1, L2) genes, URR, and forward (red arrows) and reverse (blue arrows) HPV31 primers is indicated below the genomic positions.

**a b**

**
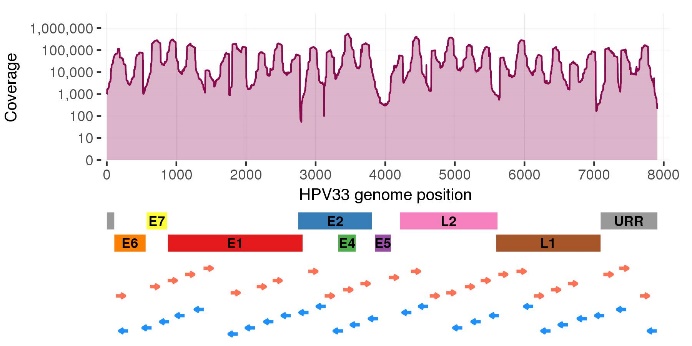

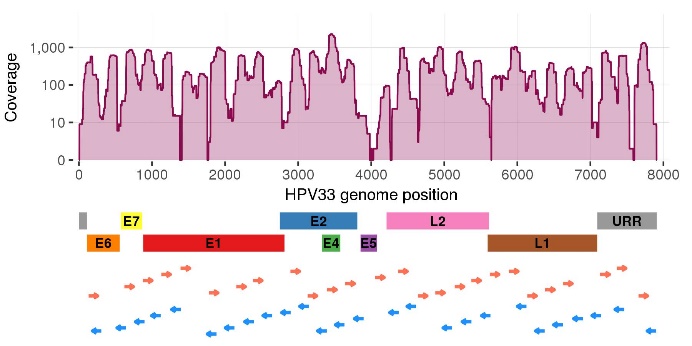
**

**c d**


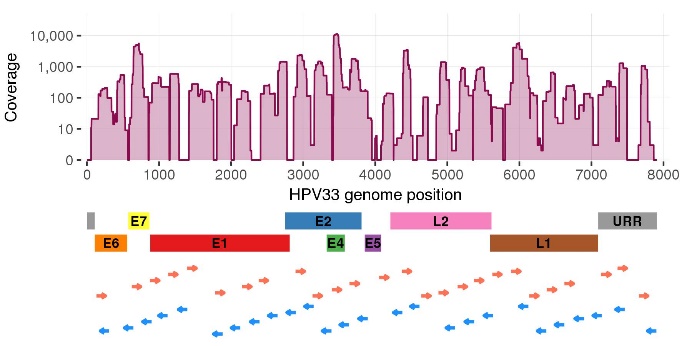

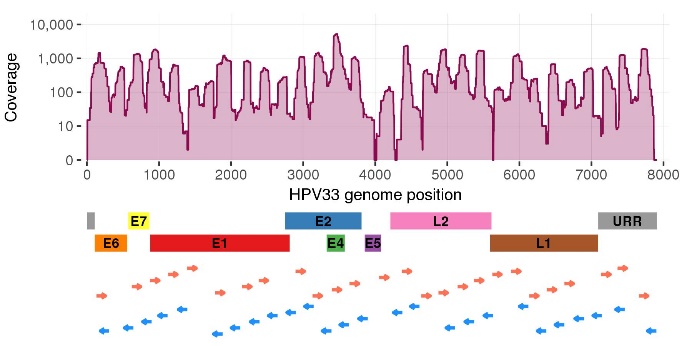


**e**


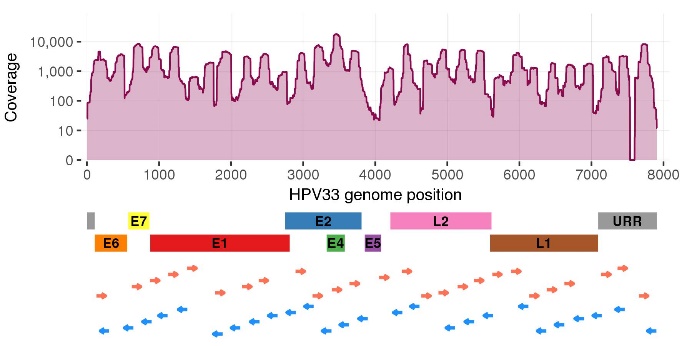


**Supplementary Figure S4.** HPV genome sequencing coverage of HPV33 positive samples a) HPV33 plasmid, b) LBC30, c) LBC31, d) LBC52, and e) LBC65. The coverage plots are aligned to the HPV33 genome. Location of early (E1, E2, E4-7), late (L1, L2) genes, URR, and forward (red arrows) and reverse (blue arrows) HPV33 primers is indicated below the genomic positions.

**a b**

**
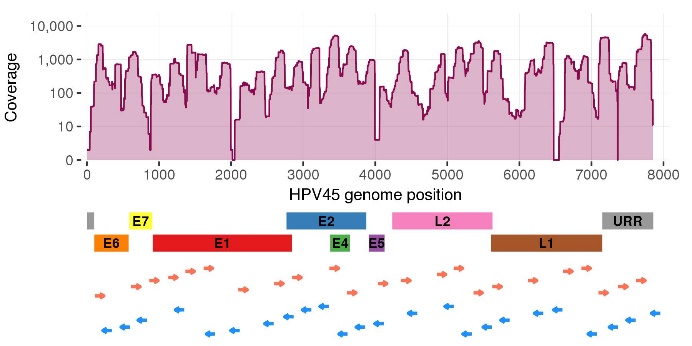

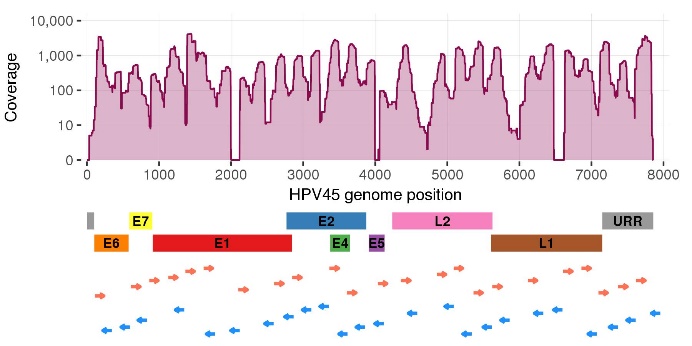
**

**c d**


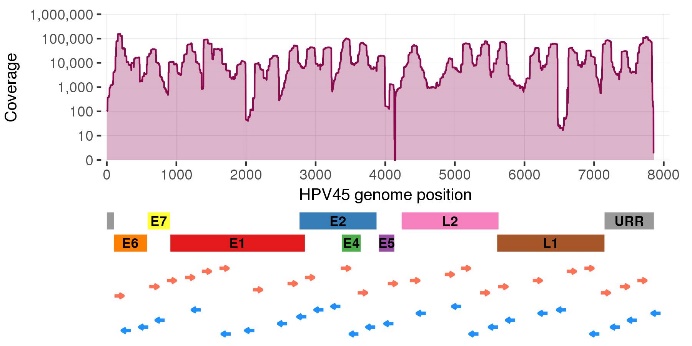

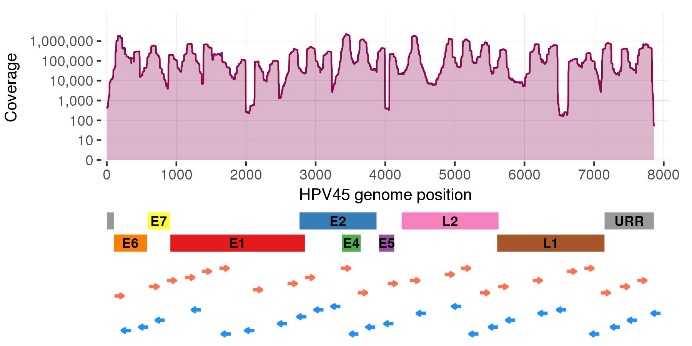


**e**


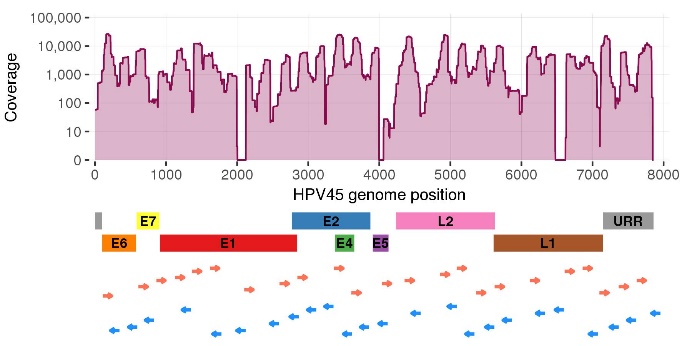


**Supplementary Figure S5.** HPV genome sequencing coverage of HPV45 positive samples a) LBC13, b) LBC29, c) LBC36, d) LBC54, and e) LBC64. The coverage plots are aligned to the HPV45 genome. The location of early (E1, E2, E4-7), late (L1, L2) genes, URR, and forward (red arrows) and reverse (blue arrows) HPV45 primers is indicated below the genomic positions.

**a b**

**
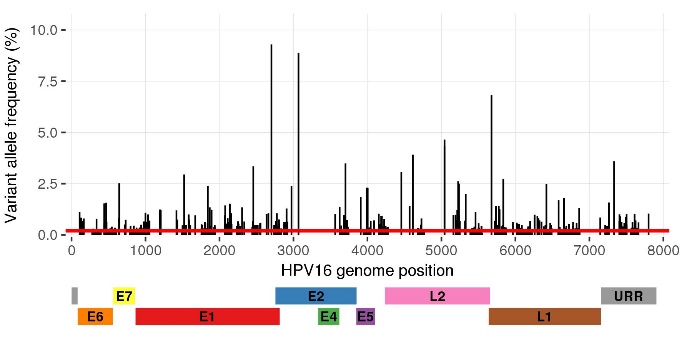

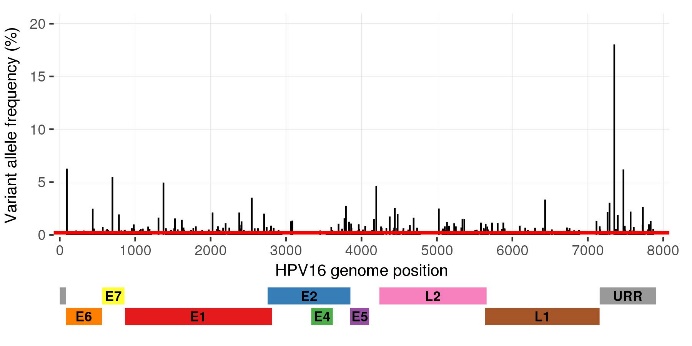
**

**c d**


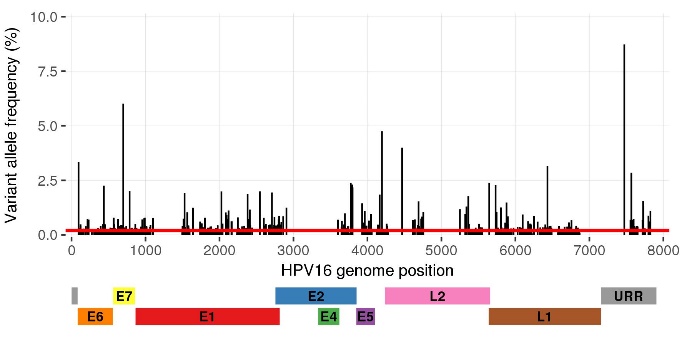

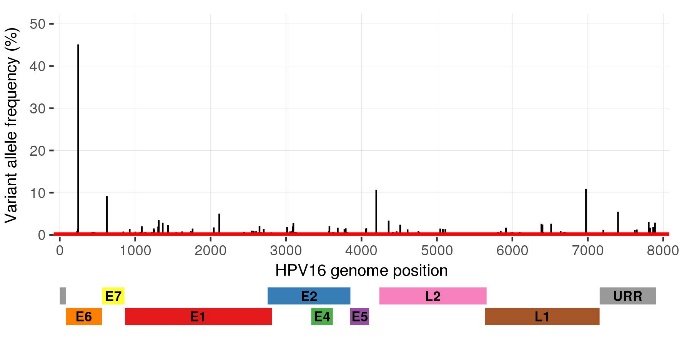


**e f**


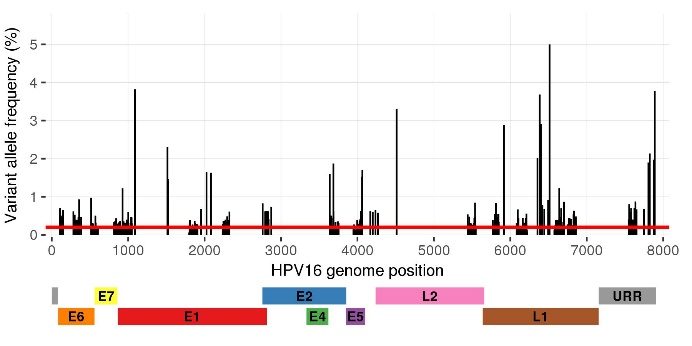

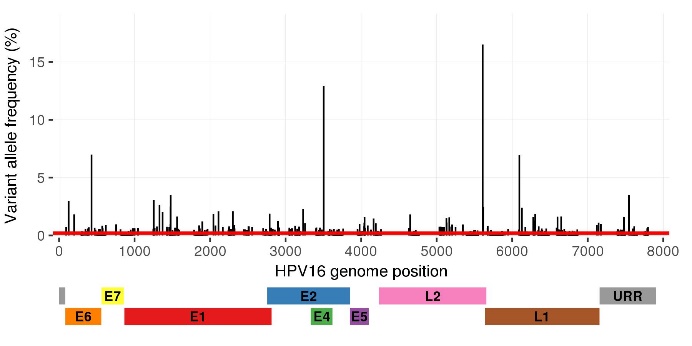


**g**


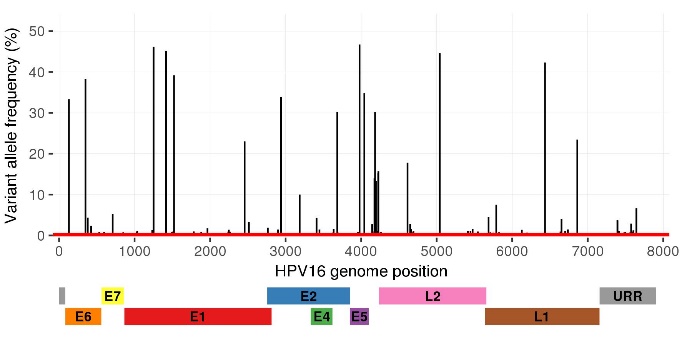


**Supplementary Figure S6.** Variable sites and variant allele frequency (%) in HPV16 positive samples a) SiHa (sequenced on MiSeq), b) SiHa-1 (sequenced on HiSeq), c) SiHa-1 (sequenced on MiSeq), d) SiHa-2 (sequenced on HiSeq), e) SiHa-2 (sequenced on MiSeq), f) LBC1, and g) LBC7. The variant plots are aligned to the HPV16 genome with the location of genes and URR. The red line indicates the variant calling threshold value of 0.2%.

**a b**

**
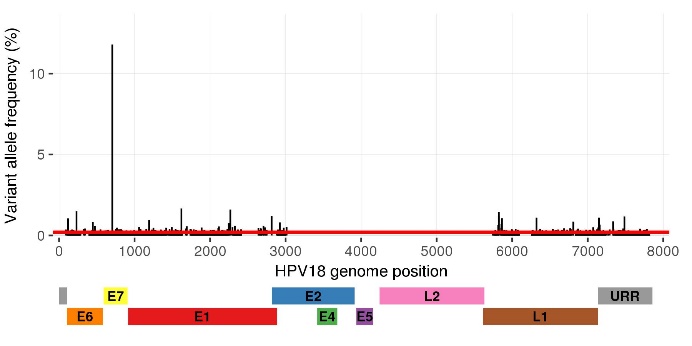
**
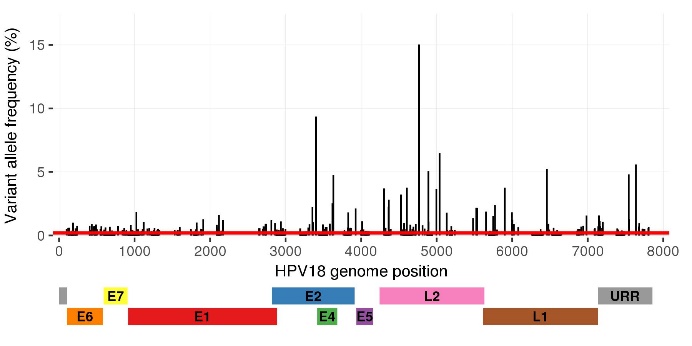


**c d**


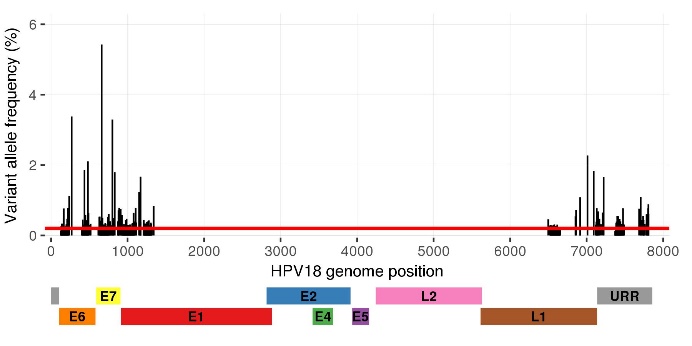

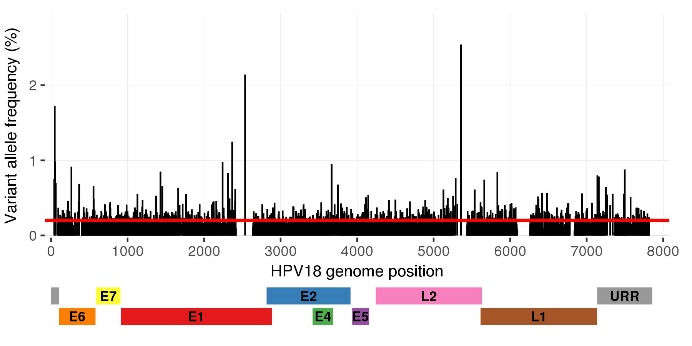


**e f**


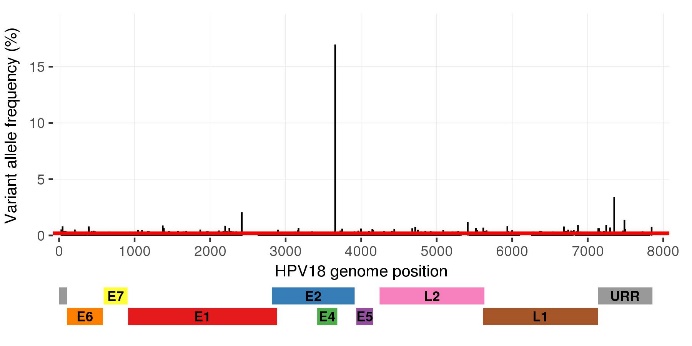

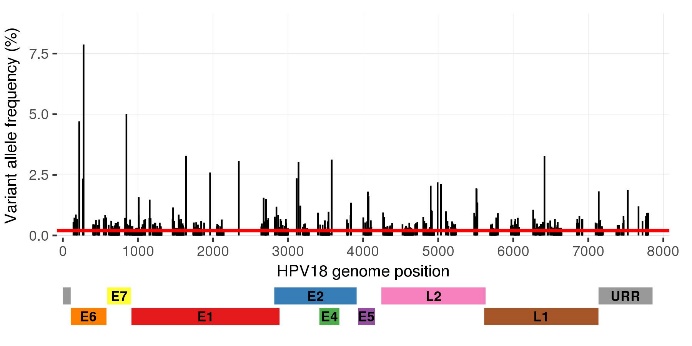


**Supplementary Figure S7.** Variable sites and variant allele frequency (%) in HPV18 positive samples a) HeLa, b) LBC103, c) LBC105, d) LBC107, e) LBC108, and f) LBC48. The variant plots are aligned to the HPV18 genome with the location of genes and URR. The red line indicates the variant calling threshold value of 0.2%.

**a b**

**
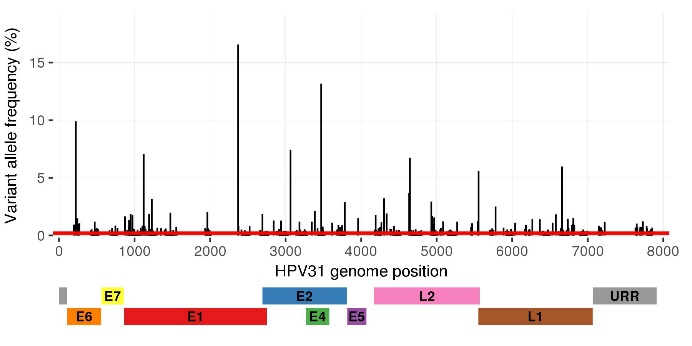

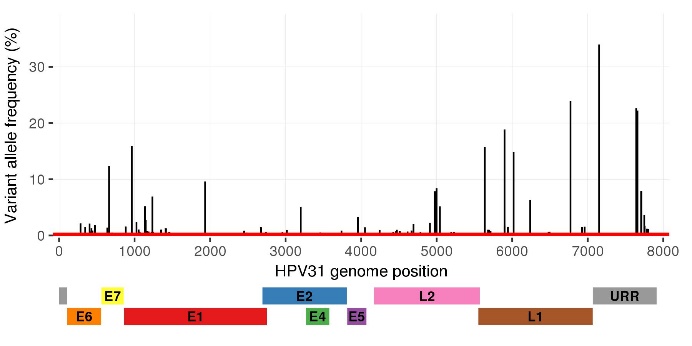
**

**c d**


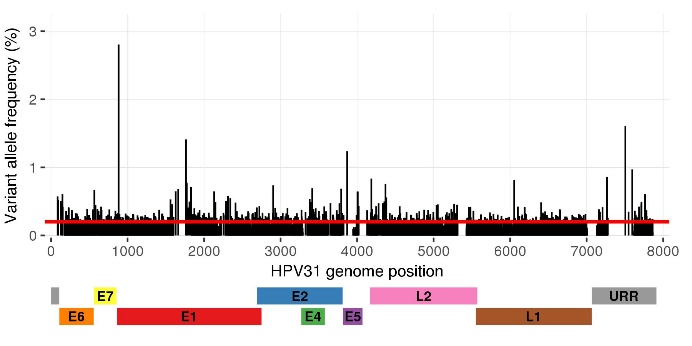

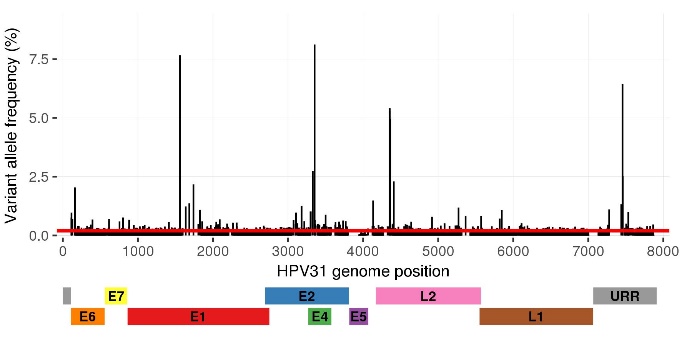


**Supplementary Figure S8.** Variable sites and variant allele frequency (%) in HPV31 positive samples a) LBC16, b) LBC24, and c) LBC32, and d) LBC34. The variant plots are aligned to the HPV31 genome with the location of genes and URR. The red line indicates the variant calling threshold value of 0.2%.

**a b**

**
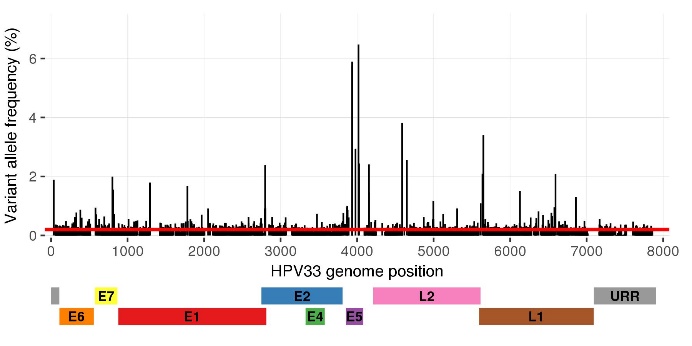

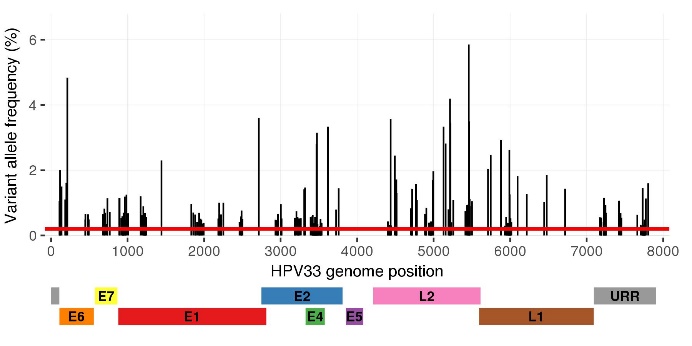
**

**c d**


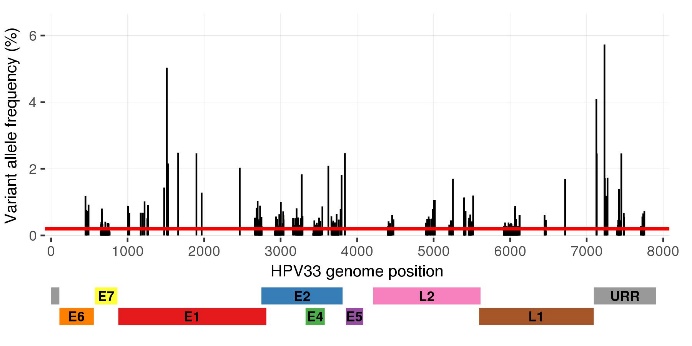

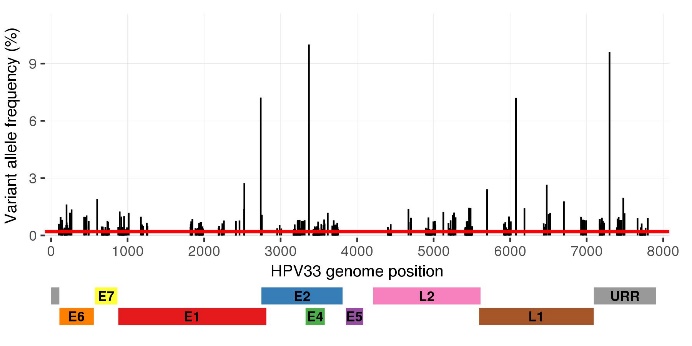


**e**


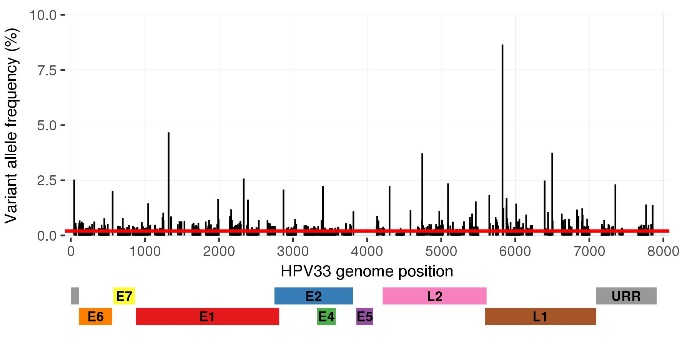


**Supplementary Figure S9.** Variable sites and variant allele frequency (%) in HPV33 positive samples a) LBC11, b) LBC30, c) LBC31, d) LBC52, and e) LBC65. The variant plots are aligned to the HPV33 genome with the location of genes and URR. The red line indicates the variant calling threshold value of 0.2%.

**a b**

**
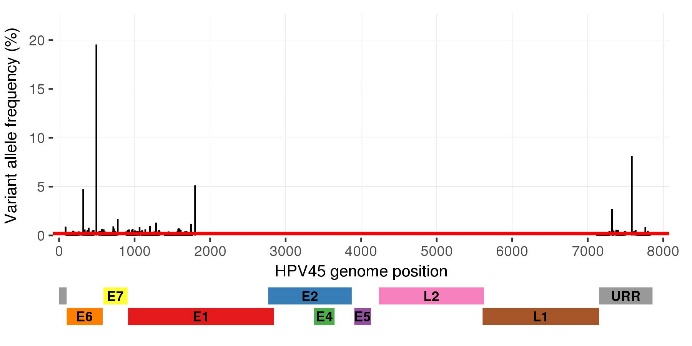

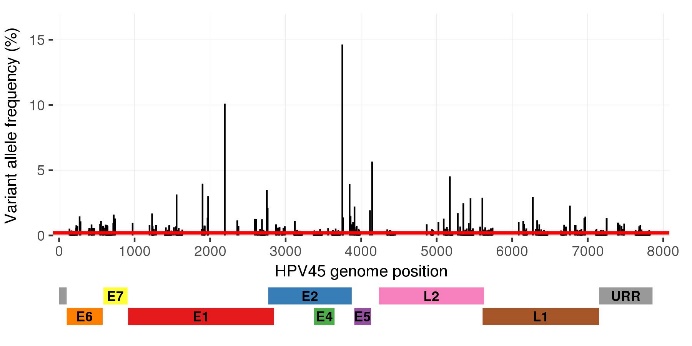
**

**c d**


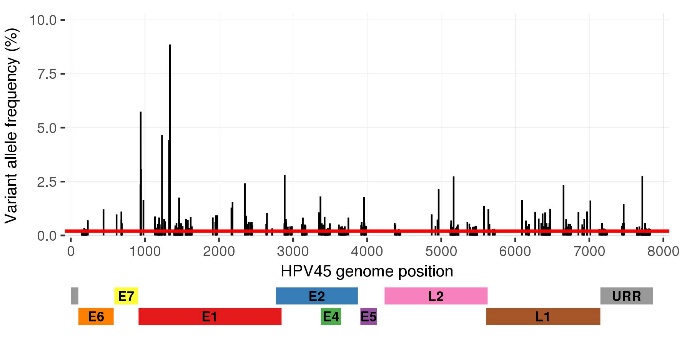

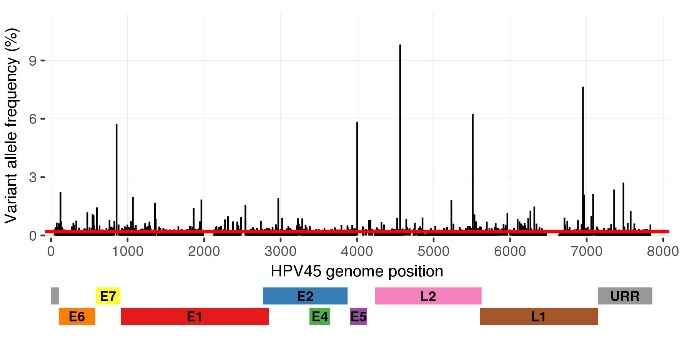


**e**


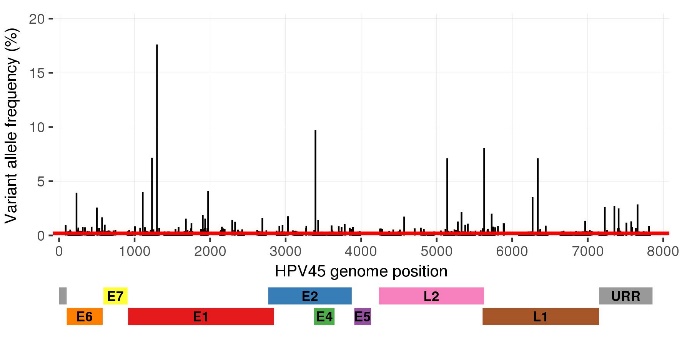


**Supplementary Figure S10.** Variable sites and variant allele frequency (%) in HPV45 positive samples a) MS751, b) LBC13, c) LBC29, d) LBC36, and e) LBC64. The variant plots are aligned to the HPV45 genome with the location of genes and URR. The red line indicates the variant calling threshold value of 0.2%.
